# Cellular and Microcircuit Mechanisms of Anesthetic Disruption of Conscious-State Organization

**DOI:** 10.3390/cells15161486

**Published:** 2026-08-18

**Authors:** Bernard Kordas

**Affiliations:** Department of Human Physiology and Pathophysiology, School of Medicine, Collegium Medicum, University of Warmia and Mazury, 10-082 Olsztyn, Poland; bernard.kordas@uwm.edu.pl

**Keywords:** general anesthesia, consciousness, pyramidal neurons, apical dendrites, thalamocortical loops, ketamine, propofol, neuronal complexity, cortical hierarchy, reportability

## Abstract

**Highlights:**

**What are the main findings?**

- Anesthesia usually disrupts neural coordination without globally silencing the brain. Local firing and sensory responses may persist even when dendritic integration, recurrent processing, cortical feedback, and thalamocortical communication are impaired.
- Propofol and volatile agents mainly restrict distributed processing. Ketamine reorganizes neural activity in a manner that changes with dose and may permit substantial activity and internally generated experience to persist.

**What are the implications of the main findings?**

- Lack of behavioral response or postoperative recall cannot directly establish the absence of conscious experience, because responsiveness, connectedness, memory, reportability, and conscious content can become dissociated.
- Assessment of anesthetic states should be adapted to each agent and integrate cellular and circuit mechanisms with thalamocortical dynamics, glial regulation, memory function, and activity across the brain.

**Abstract:**

The reversible effects of general anesthesia can be used to examine how brain activity changes during transitions into and out of altered conscious states. Such changes are commonly tracked using behavioral responses, electroencephalographic recordings, and measures of functional connectivity. These measures, however, reflect processes occurring at lower levels of organization, including the activity of pyramidal neurons, dendritic integration, interneuron function, thalamocortical signaling, and glial regulation of the extracellular environment. Anesthesia does not affect all neural processes equally. Propofol and volatile agents interfere with synaptic transmission and apical dendritic integration, as well as cortical feedback and thalamocortical signaling. The resulting activity is not necessarily absent or uniformly weaker. It is often more stereotyped, temporally restricted, and poorly coordinated between regions. Ketamine produces a different organization. Substantial neural activity and complex cortical responses may persist after behavioral responsiveness has been lost, although deeper anesthesia also produces slower and less complex activity. Responsiveness, environmental connectedness, memory, reportability, and conscious content may therefore become partly uncoupled. Ketamine produces a dissociative state that cannot be interpreted simply as another form of the predominantly restrictive state produced by propofol or volatile anesthetics. This narrative review synthesizes evidence identified through a structured PubMed search to examine how anesthesia reshapes conscious processing and why unresponsiveness or absent recall may not indicate absent experience.

## 1. Introduction

General anesthesia provides a reversible means of examining how the brain enters and leaves states in which responsiveness and contact with the environment are altered. Here, conscious-state organization refers to the joint configuration of neural integration, behavioral responsiveness, environmental connectedness, conscious content, memory, and reportability. Anesthetic exposure can be adjusted while behavior and neural activity are followed during induction and recovery, without the structural damage associated with brain injury. These transitions are not governed by drug concentration alone. Experiments in insects and mammals have demonstrated hysteresis between induction and emergence, while human recordings show that responses to stimuli of different salience disappear and return in a different order. Around the loss of responsiveness, small increases in propofol concentration can also produce abrupt changes in network organization [1,2,3].

Loss of behavioral response does not imply complete inhibition of brain activity. During propofol anesthesia, connectivity within frontoparietal and higher-order thalamocortical networks declines, while connectivity in low-level auditory and visual systems can remain relatively preserved [4]. Intracranial recordings show that local cortical populations retain short-range patterns of activity and can continue to fire during restricted phases of a slow oscillation, although their activity becomes fragmented across time and cortical regions [5]. Slow cortical-potential networks and cross-frequency coupling may persist even during deeper anesthesia [6]. The presence of local activity or a residual network structure is therefore insufficient to establish preserved conscious access or distributed integration when communication between regions has become temporally restricted or poorly coordinated.

The major behavioral and cognitive endpoints of anesthesia do not always change together. Amnesia can be experimentally distinguished from sedative and hypnotic effects by the involvement of GABA(A) receptors that contain the α5 subunit in the hippocampus [7]. Ketamine provides a different example. Participants rendered unresponsive with ketamine retained complex cortical responses to stimulation and later reported vivid experiences, unlike participants exposed to propofol or xenon at a comparable behavioral endpoint [8]. Studies using serial awakenings during sedation and sleep have also identified differences between sensory disconnection and the absence of reported experience. EEG measures previously interpreted as markers of unconsciousness were often associated with environmental connectedness as well, and none of the measures examined was specific only to the presence or absence of conscious experience [9,10]. In a randomized study of 160 healthy men, thalamic metabolism likewise distinguished connected from disconnected states under propofol, dexmedetomidine, and sevoflurane, but not under S-ketamine [11].

Similar behavioral endpoints can consequently arise from different forms of neural organization. Propofol limits the size and duration of neuronal avalanches and reduces the complexity of cortical dynamics, whereas ketamine allows more wake-like activity to persist [12]. Cortical responses to electrical stimulation become simpler during propofol and sevoflurane anesthesia, whereas the effects of ketamine depend on anesthetic depth and the cortical region examined [13]. Across the three anesthetics, loss of reported experience coincided with departures from critical avalanche behavior and from dynamics near the transition to chaos. Ketamine nevertheless allowed vivid experience to persist despite behavioral unresponsiveness [14]. Unresponsiveness should therefore be treated as an observation about behavior, not as a complete description of the underlying state.

The present review examines how these macroscopic differences arise from cellular and microcircuit operations. Attention is given to synaptic transfer, apical dendritic integration, pyramidal-cell output, interneuron activity, recurrent cortical processing, thalamocortical communication, memory mechanisms, and regulation of the neural microenvironment by glial cells. Experimental studies show that anesthesia can uncouple distal apical dendritic activity from somatic firing, synchronize layer 5 pyramidal neurons across large cortical distances, and suppress astrocytic calcium signaling [15,16,17]. Propofol and volatile anesthetics are considered mainly as models in which access to wider cortical processing is constrained. Ketamine is examined as a contrasting condition in which substantial activity persists but is reorganized, separating responsiveness from connectedness, memory, reportability, and conscious content. The aim is to trace how changes within individual cells and local circuits develop into distinct anesthetic states.

### Methodology

PubMed was searched using seven title/abstract search modules designed to cover the main thematic axes of the review: general anesthesia as a perturbation of consciousness (S1); cellular, dendritic, pyramidal-neuron, synaptic, and intracellular molecular mechanisms (S2); propofol and volatile anesthetic effects on electroencephalographic activity, synchrony, feedback, and predictive processing (S3); thalamocortical and corticothalamocortical mechanisms of loss and recovery of consciousness (S4); ketamine, dissociation, cortical complexity, and memory (S5); reportability, memory, and connectedness under anesthesia (S6); and astrocytic and glial modulatory mechanisms (S7). Appendix A Table A1 provides the exact queries used in these modules. Two PubMed filters were applied to these queries: a publication date range from 1 January 2005 to 1 July 2026 and English language. The final search used the seven modules as a single PubMed query separated by the OR operator. In this way, each record was counted only once—even if multiple modules retrieved it.

The titles of 2401 records were screened for potential relevance to cellular, microcircuit, thalamocortical, corticocortical, glial, memory, reportability, or dissociative mechanisms involved in anesthetic disruption of conscious-state organization. The retained title set was reviewed a second time before abstract screening to ensure consistency of inclusion. Records with uncertain relevance were retained at this stage to preserve sensitivity before abstract screening. In total, 778 records remained for abstract evaluation, which retained experimental, neurophysiological, imaging, computational, theoretical, and review articles, including meta-analyses that addressed anesthetic effects on consciousness, responsiveness, connectedness, memory, reportability, or cellular-to-system integration. This evaluation yielded 119 articles discussed in this review. All title and abstract screening was conducted by one author. The second title review was a consistency check by the same author and not an independent assessment. The full texts of the retained articles were consulted during synthesis; no separate full-text exclusion stage was conducted.

Because this is a narrative review, article selection emphasized conceptual and mechanistic relevance rather than formal study ranking or quantitative synthesis. The modules are relatively specific combinations of title and abstract terms to keep the final record set manageable within the interdisciplinary scope of the review, which included anesthesiology, consciousness science, systems neuroscience, cellular mechanisms, glial biology, and computational approaches. As a result, relevant studies using different terminology or studies that did not explicitly include the selected descriptors in the title or abstract may not have been retrieved.

## 2. Cellular and Microcircuit Conditions for Conscious Processing

### 2.1. Apical Dendrites and Pyramidal-Neuron Integration

Neuronal firing alone does not account for conscious processing. Cortical neurons must also integrate sensory signals from lower levels with contextual influences, cortical feedback, thalamic input, and neuromodulation. Neocortical pyramidal cells are anatomically adapted to perform this function because separate pathways can deliver information to different parts of their extensive dendritic tree.

In one study, it was proposed that pyramidal neurons contribute to the maintenance of complex and sustained patterns of activity within thalamocortical circuits [18]. From this perspective, the relevant issue is not simply whether cortical neurons remain active, but whether signals can be combined across cellular compartments and organizational scales. Local activity may therefore continue during unconscious or disconnected states without acquiring the stability and wider influence required for conscious processing. Apical dendrites provide a central substrate for this form of integration. Models of anesthetic suppression propose that conscious perception requires effective communication between basal sensory input and apical dendritic signals that carry contextual or feedback information [19]. Related theoretical work on apical amplification suggests that arousal and top-down modulation may selectively amplify contextually relevant signals in neocortical pyramidal cells [20]. Within this model, disruption of consciousness can arise when subcellular mechanisms of selective amplification fail, even if local excitability is not abolished.

Experiments in the mouse cortex provide direct evidence that anesthesia alters communication between apical dendrites and the soma of layer 5 pyramidal neurons [15]. Distal dendritic depolarization produced strong somatic firing in awake animals, but this effect disappeared under anesthesia. Neuromodulatory signaling and input from higher-order thalamic nuclei were also necessary for effective coupling. Apical integration should therefore be understood as part of a wider corticothalamic arrangement and not a mechanism confined to an individual neuron. The position of layer 5 pyramidal neurons makes them particularly important: they receive signals from several cortical layers and long-range pathways while providing a major route of cortical output. In vivo two-photon calcium imaging in mice (nine awake and eight per anesthetic condition) showed that general anesthesia produced widespread synchronization of spontaneous activity specifically in layer 5 pyramidal neurons, an effect that was not consistently observed in other cortical cell types [16]. Disruption of the conscious state may consequently reflect an abnormal temporal organization of pyramidal activity, together with altered communication between dendritic compartments and somatic output.

These findings support a cellular model in which conscious processing depends on flexible integration within pyramidal neurons, especially through apical dendrites and layer 5 output systems. Local spiking alone is therefore insufficient as a marker of conscious access. The critical issue is whether cellular activity remains dynamically coupled to the dendritic, thalamocortical, and microcircuit processes that allow it to influence an integrated conscious state.

### 2.2. Interneurons, Excitation–Inhibition Balance, and Recurrent Processing

Within the cortex, pyramidal neurons function as elements of local circuits, and not in isolation. Their activity is continually shaped by interneuron subtypes, recurrent excitatory connections, neuromodulatory systems, and the organization of individual cortical layers. Area and layer specificity are important features of this organization. Recordings from ferret visual and prefrontal cortex under isoflurane anesthesia show that cortical regions and laminar compartments differ in their vulnerability to anesthetic modulation, with association cortex showing broader spectral-power changes than primary visual cortex [21]. Two-photon imaging in the mouse barrel cortex indicates that deep anesthesia can impose local neuronal synchrony while disrupting spatially organized sensory processing [22]. Information-theoretic analyses add that anesthetic modulation of active information storage is specific to frequency and layer, with gamma-band effects concentrated mainly in superficial layers and alpha/beta changes more prominent in deeper layers [23]. These findings suggest that conscious processing depends on a structured laminar and spectral organization.

Excitation–inhibition balance is likewise reorganized differently across cell types. In a chronic in vivo two-photon study of the mouse visual cortex, with three to seven mice per cell-type comparison, 1.2% isoflurane was associated with reduced cortical GABA transmission, relatively preserved glutamate transmission onto pyramidal cells, and reduced glutamate transmission onto inhibitory interneurons. PV and VIP interneurons followed different trajectories: PV cells became less active and more synchronized, whereas changes in VIP activity appeared later [24]. This pattern challenges a simple model in which anesthesia causes unconsciousness only by increasing inhibition. Instead, the local cortical state may become less capable of flexible integration because inhibitory and excitatory subpopulations are differentially reorganized. Comparative, in vivo two-photon imaging of excitatory neurons and interneurons expressing PV and SST, with eight mice per neuronal population, indicates that different anesthetics can produce a similar degree of sedation through different local circuit changes. Isoflurane, propofol, and ketamine reduced population activity in both excitatory and inhibitory cells at comparable behavioral depths. Ketamine, however, increased activity in a subset of excitatory neurons and preferentially recruited SST interneurons [25]. Comparable behavioral suppression, therefore, cannot be assumed to correspond to an equivalent microcircuit configuration.

Recurrent activity provides another condition for conscious processing. In medial prefrontal cortex slices, muscarinic activation can induce long-lasting plateau potentials and sustained spiking in layer 2/3 pyramidal neurons, resembling aroused cortical dynamics [26]. Propofol suppresses these plateau potentials and associated spiking, whereas ketamine produces partially divergent effects, which depend on dose. Such findings indicate that anesthetics act not only on momentary synaptic transmission, but also on the cellular substrates of sustained recurrent activity.

**Figure 1:**
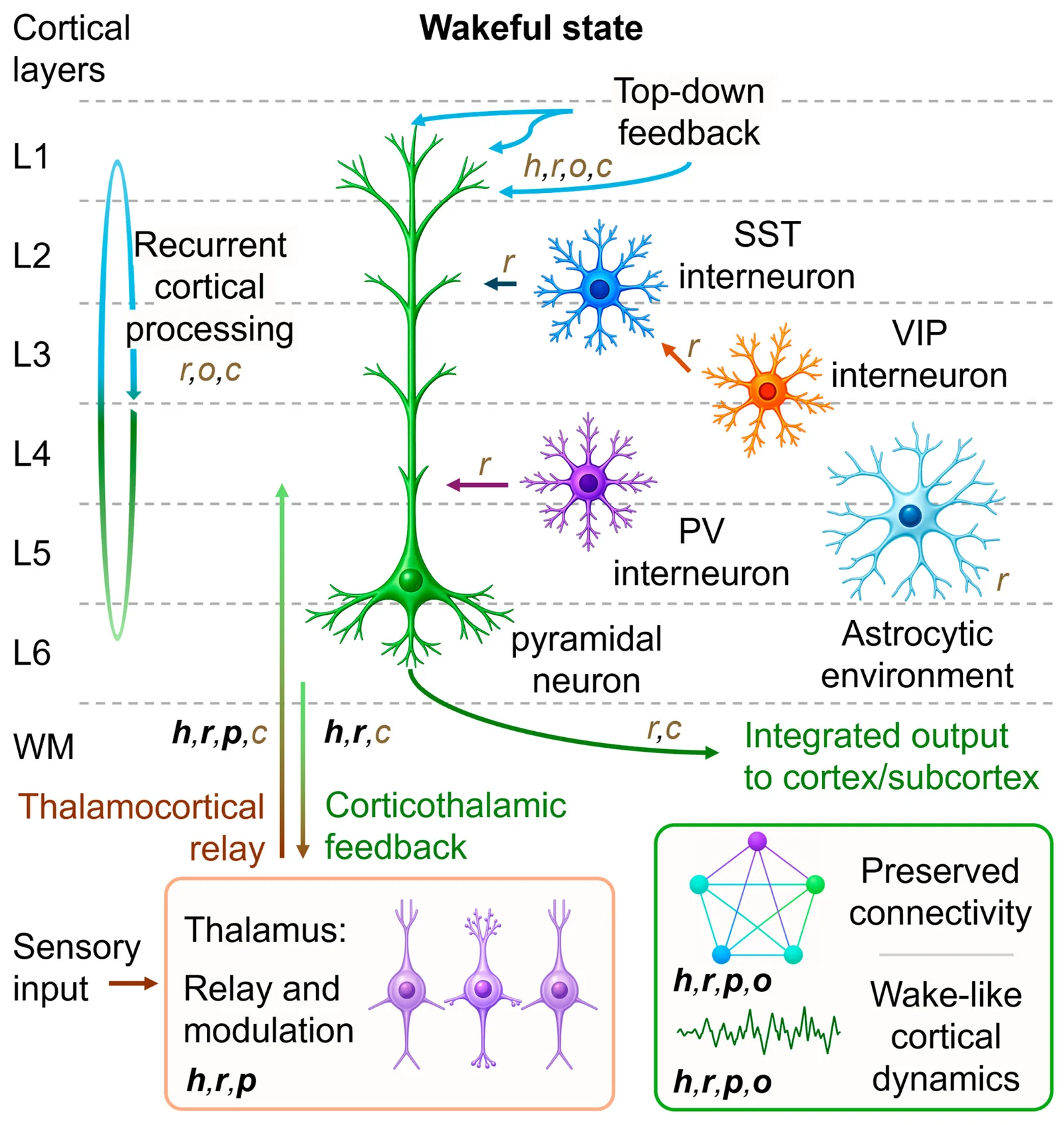
Cellular and microcircuit organization supporting the wakeful integrated state. A cortical pyramidal neuron integrates thalamocortical input with local recurrent processing and top-down feedback arriving at its apical dendrites. Interactions among pyramidal neurons and somatostatin-expressing (SST), vasoactive intestinal peptide-expressing (VIP), and parvalbumin-expressing (PV) interneurons provide dendritic, disinhibitory, and perisomatic regulation of cortical activity, respectively. Bidirectional communication between the cortex and thalamus comprises ascending thalamocortical relay and descending corticothalamic feedback, while astrocytes contribute to regulation of the local cellular environment. Together, these mechanisms support integrated cortical and subcortical output, preserved network connectivity, and integrated cortical dynamics characteristic of wakefulness. The figure is a conceptual representation and does not depict a complete wiring diagram. Evidence codes identify the source and level of support for individual elements: bold italic black codes indicate direct experimental support for the labeled process, while ochre italic codes indicate indirect or partial support, or inference from related measurements or models. Arrow colors distinguish pathway classes or the associated cell type and do not represent evidence strength. The multicolored recurrent loop represents processing across cortical layers; its color transitions do not indicate different evidence levels. *h*, human studies; *r*, rodent studies; *p*, non-human primate studies; *o*, studies in other species; *c*, computational or theoretical studies; L1–L6, cortical layers 1–6; WM, white matter; SST, somatostatin-expressing interneuron; VIP, vasoactive intestinal peptide-expressing interneuron; PV, parvalbumin-expressing interneuron.

Together, these studies indicate that conscious processing depends on coordinated interactions among pyramidal neurons, interneuron subtypes, recurrent excitation, laminar organization, and frequency information processing. General anesthetics perturb these conditions in different ways, reducing the capacity of local microcircuits to support flexible, hierarchically integrated, and context-sensitive processing. These cellular and microcircuit conditions are summarized as a schematic wakeful reference state in Figure 1. With this reference established, classical anesthetics can be examined as perturbations that constrain conscious access by disrupting the same integrative operations.

## 3. Classical Anesthetics as Suppressors of Cellular-to-Network Integration

Propofol and volatile anesthetics are often defined by the behavioral endpoint of lost responsiveness. At the cellular and circuit levels, however, their effects are more specific. Sensory responses, local spiking, and elements of large-scale organization may persist, but neuronal activity becomes less able to support flexible, hierarchically integrated, and reportable conscious access. This section examines suppression across three linked levels: higher-order network integration, synaptic and pyramidal-neuron mechanisms, and the dynamical constraints imposed on cortical activity.

### 3.1. Propofol and Volatile Anesthetics as Models of Suppressed Conscious Access

Human neuroimaging and electrophysiological studies indicate that propofol mainly disrupts higher-order integration, instead of abolishing all neural activity. In healthy volunteers, propofol reduced corticocortical and thalamocortical connectivity in frontoparietal, default-mode, and executive-control networks, while low-level auditory and visual networks showed relative preservation [4]. Conscious access may therefore fail when sensory activity can no longer be integrated with associative and thalamocortical systems. Intracranial recordings support this interpretation at finer temporal and spatial scales. Behavioral responsiveness disappeared rapidly after a field-potential rhythm below 1 Hz emerged during propofol induction. Yet, local cortical populations retained millisecond-scale connectivity, and spiking remained possible during restricted phases of the oscillation [5]. The relevant transition was not the disappearance of local activity, but the fragmentation of its timing and coordination across cortical areas.

Propofol can also alter cortical dynamics while preserving parts of large-scale organization. Human ECoG recordings during induction and emergence showed that slow cortical-potential networks and theta–gamma phase-power coupling could persist even during deep burst suppression [6]. Preserved anatomical or low-frequency architecture is therefore not sufficient for conscious access when flexible interareal coordination is lost. During propofol induction, responses to less salient click stimuli disappeared before responses to verbal stimuli. The corresponding recovery sequence and EEG changes are described in Section 4.3 [2]. This pattern indicates that behavioral responsiveness reflects the interaction among anesthetic depth, stimulus properties and the current organization of cortical activity.

Comparable evidence from nonhuman primates suggests that suppression of access unfolds as a sequence of discrete neural events. In two macaques trained to keep one hand on a button until the end of each trial to receive a liquid reward, coherent beta oscillations between the primary somatosensory cortex and the ventral premotor cortex were disrupted before loss of behavioral responsiveness; after this transition, neurons shifted from bimodal tactile-auditory responses to predominantly tactile responses [27]. This supports a model in which propofol progressively limits multisensory and associative integration.

Baseline network organization also influences susceptibility to propofol. In humans, weaker alpha-band networks before sedation predicted later unresponsiveness despite similar blood propofol levels, while slow–alpha phase-amplitude coupling tracked drug concentration [28]. Drug concentration, intrinsic network susceptibility, and the presence or absence of an observable response therefore represent related, but not interchangeable, features of an anesthetic state.

Across these studies, sensory responses and local neuronal activity were not necessarily abolished. Their participation in higher-order coordination was instead restricted. The following section considers how synaptic transmission, dendritic coupling, and pyramidal-cell output may generate this disruption at the macroscopic level.

### 3.2. Synaptic and Dendritic Constraints on Pyramidal-Neuron Output

In the ventrobasal thalamus of mice, propofol altered the response of relay neurons to excitatory input. At clinically relevant concentrations, it reduced EPSP summation and spike generation, decreased input resistance, and prolonged inhibitory currents through GABA(A) receptors while increasing their amplitude [29]. These changes are consistent with stronger shunting inhibition and less effective transmission of sensory activity.

Isoflurane produced a related change in thalamic somatosensory processing. High concentrations converted sustained whisker-evoked responses in the rat ventral posteromedial thalamus into brief ON responses. Local iontophoretic application of NMDA or AMPA receptor agonists, or GABA(A) receptor antagonists, reversed the suppression of sustained responses produced by higher isoflurane concentrations in most neurons [30]. Whisker-evoked input still reached the thalamus, although the sustained component of the response was lost before effective transmission to the cortex. Etomidate reduced discharge in mouse thalamocortical neurons through effects on both synaptic and extrasynaptic GABA(A) receptors. Tonic inhibition contributed substantially to this response [31]. In vivo enhancement of thalamic spillover inhibition mediated by extrasynaptic GABA(A) receptors has also been linked to an electrocortical signature of hypnosis [32]. Impaired thalamic relay can consequently arise from both sustained and synaptic forms of inhibition. A related effect occurs in cortical pyramidal neurons. General anesthetics uncoupled distal apical dendritic activity from somatic output, so that apical depolarization no longer produced the strong firing observed in the awake state [15]. Contextual and feedback input may reach the apical dendrites without exerting its usual effect on cortical output.

General anesthetics also act on presynaptic release machinery, a class of targets that can connect molecular binding to circuit output [33]. Volatile anesthetics bind synaptic SNARE proteins, and isoflurane, etomidate, and propofol inhibit release machinery through partly distinct sites [34,35,36]. Syntaxin1A mutations also alter isoflurane resistance and recovery in Drosophila, linking presynaptic release to a behavioral anesthetic endpoint without establishing the same mechanism in humans [37].

Changes in layer 5 activity extend this mechanism from neuronal compartments to population organization. Across several general anesthetics, spontaneous activity in mouse layer 5 pyramidal neurons became selectively synchronized over large cortical distances. Other cortical cell types did not display the same consistent response [16]. Excessive synchronization can narrow the range of pyramidal output and place cortical populations in a less adaptable activity regime. Chemogenetic activation replaced slow-wave activity with tonic firing within the targeted hemisphere, but behavioral responsiveness did not return [38]. During perfusion with approximately 22 μM sevoflurane, layer 5 pyramidal neurons from the mouse primary auditory cortex gradually lost their capacity for repetitive firing. The addition of 100 nM TsTX-Kα partially restored selected subthreshold currents, but neuronal firing remained below control levels. Kv1.2 modulation could thus account for part, though not all, of the reduction in excitability [39]. In a separate study of the medial prefrontal cortex, sevoflurane reduced pyramidal-cell firing. Silencing this neuronal population increased sensitivity to volatile anesthesia, while its activation had the opposite effect [40].

Propofol also changes the relationship between neuronal firing and local field activity. In human and feline intracortical recordings, increasing concentrations reduced high-frequency local field potential power and action potential firing rates while generating large low-frequency spike-like events [41]. As propofol concentration increased, spikes occurred within a narrower phase window of the slow local field potential. This left less temporal freedom for firing-rate coding. The effects appeared at several levels: thalamic relay, apical coupling, layer 5 population timing, intrinsic excitability, and spike–field timing. Each change narrowed the routes through which local activity could shape cortical output.

Taken together, the role of GABA(A) signaling differs across anesthetic agents, receptor subtypes, and brain regions. In the hippocampus, α5-containing receptors contribute to the amnestic effect of etomidate but do not account for its principal sedative action. In thalamic relay neurons, synaptic and extrasynaptic GABA(A) currents contribute to suppression produced by propofol, etomidate, and volatile anesthetics. In the cortex, isoflurane can reduce GABA transmission while altering the activity and synchronization of PV and VIP interneurons. Ketamine follows a different pattern. Its effects cannot be explained by generalized enhancement of GABA signaling and instead involve altered interneuron recruitment after NMDA receptor drive is reduced. Potentiation of inhibition is therefore important for selected anesthetics and functional endpoints, but it does not constitute a single mechanism that operates throughout the brain [7,24,25,29,31,32].

### 3.3. Altered Synchrony and Reduced Flexible Dynamics

The cellular changes described above become visible at the systems level as restricted timing, simpler responses to perturbation, and transitions between distinct cortical states. During propofol anesthesia, slow oscillations confine neuronal firing to limited temporal windows. Local populations can remain active, but slow oscillations occur asynchronously across cortical regions, fragmenting communication in both time and space [5]. The loss of responsiveness is consequently accompanied by a change in how activity is coordinated, not by its complete disappearance.

Responses to direct cortical stimulation provide a measure of the remaining dynamical range. In awake rats, intracranial electrical stimulation produced activity that extended across cortical regions and frequencies: under propofol or sevoflurane, the responses contained less high-frequency activity, weaker phase locking and functional connectivity, and fewer differentiated spatiotemporal patterns [13]. Cortical networks remained responsive to stimulation, but the activity that followed was less varied and less widely coordinated.

The transition into unresponsiveness with propofol can occur abruptly. During slowly increasing propofol exposure in healthy volunteers, long-timescale connectivity based on low-frequency power envelopes showed critical slowing before a marked decline in global network efficiency, a graph measure of network-wide integration. At approximately 10 Hz, the efficiency calculated from the phase increased instead. Lower network complexity characterized both the approximately 10 Hz hypersynchronous configuration and the slower asynchronous configuration [3]. Increased synchrony and reduced connectivity can therefore occur in different frequency ranges during the same transition. Individual trajectories are not uniform. Responsiveness was associated with the direction, rather than simply the magnitude, of complexity change. Under moderate propofol sedation, Lempel–Ziv and statistical complexity shifted in opposite directions in responsive and unresponsive participants, while baseline statistical complexity predicted the subsequent trajectory [42]. A single complexity measure cannot be interpreted independently of its mathematical definition, the initial brain state, and the behavioral endpoint used in the experiment.

Criticality describes dynamics near a transition between highly ordered and highly disordered activity, where signals can propagate widely without becoming either rigidly synchronized or fragmented. Comparison of propofol, xenon, and ketamine indicated that critical dynamics were associated with reported experience, and not with unresponsiveness alone. Departure from critical avalanche statistics and from dynamics near the chaotic boundary characterized states without reported experience, while resting EEG properties covaried with perturbational complexity [14]. The findings link simplified responses to perturbation with changes already present in ongoing activity, while separating unconsciousness from unresponsiveness alone.

Deep anesthesia does not produce one fixed cortical configuration. In rats at constant desflurane concentrations, electrocortical activity spontaneously alternated between discrete states. Six states combined stronger delta activity with lower complexity and generally followed anesthetic depth. A seventh occurred during deep anesthesia but showed lower delta power and higher complexity. Transitions between states followed structured, nonrandom paths despite the unchanged anesthetic concentration [43]. Drug concentration constrains the available cortical states without determining a single pattern of activity. Burst suppression also contains organized neuronal dynamics. Cortex-wide calcium imaging and electrical recordings during isoflurane anesthesia identified different neuronal ensembles associated with bursts and suppressions. Burst onset was marked by strongly synchronized activity and high functional connectivity. Suppression periods contained more asynchronous, temporally dispersed activity and weaker connectivity. Movement between the two states followed a sequential pattern of propagation across cortical regions [44]. Alternating electrical silence and activity at the surface can thus arise from coordinated changes in the recruitment of distributed neuronal populations.

Whole-cortex imaging shows that some spatial features are shared across anesthetics, although the complete patterns remain specific to drug. Ketamine, propofol, and isoflurane all enhanced 1.5–2.5 Hz calcium oscillations in the retrosplenial cortex of mice—the power of this activity was greater in the retrosplenial cortex than in motor, somatosensory, or visual regions, while ketamine and isoflurane also produced structured signaling patterns across the cortex [45]. A common low-frequency node can therefore coexist with distinct activity distributions elsewhere. Population stability provides another point of convergence. Intracortical recordings under propofol, ketamine, and dexmedetomidine showed reduced stability of cortical dynamics despite the different molecular targets of the three agents. Neural activity recovered more slowly after sensory perturbation, and autocorrelation times associated with the stimulus became longer. The destabilization was most evident in low-frequency population activity [46]. Similar behavioral suppression can therefore emerge from a shared slowing of cortical population dynamics even when cellular and oscillatory effects differ.

**Figure 2:**
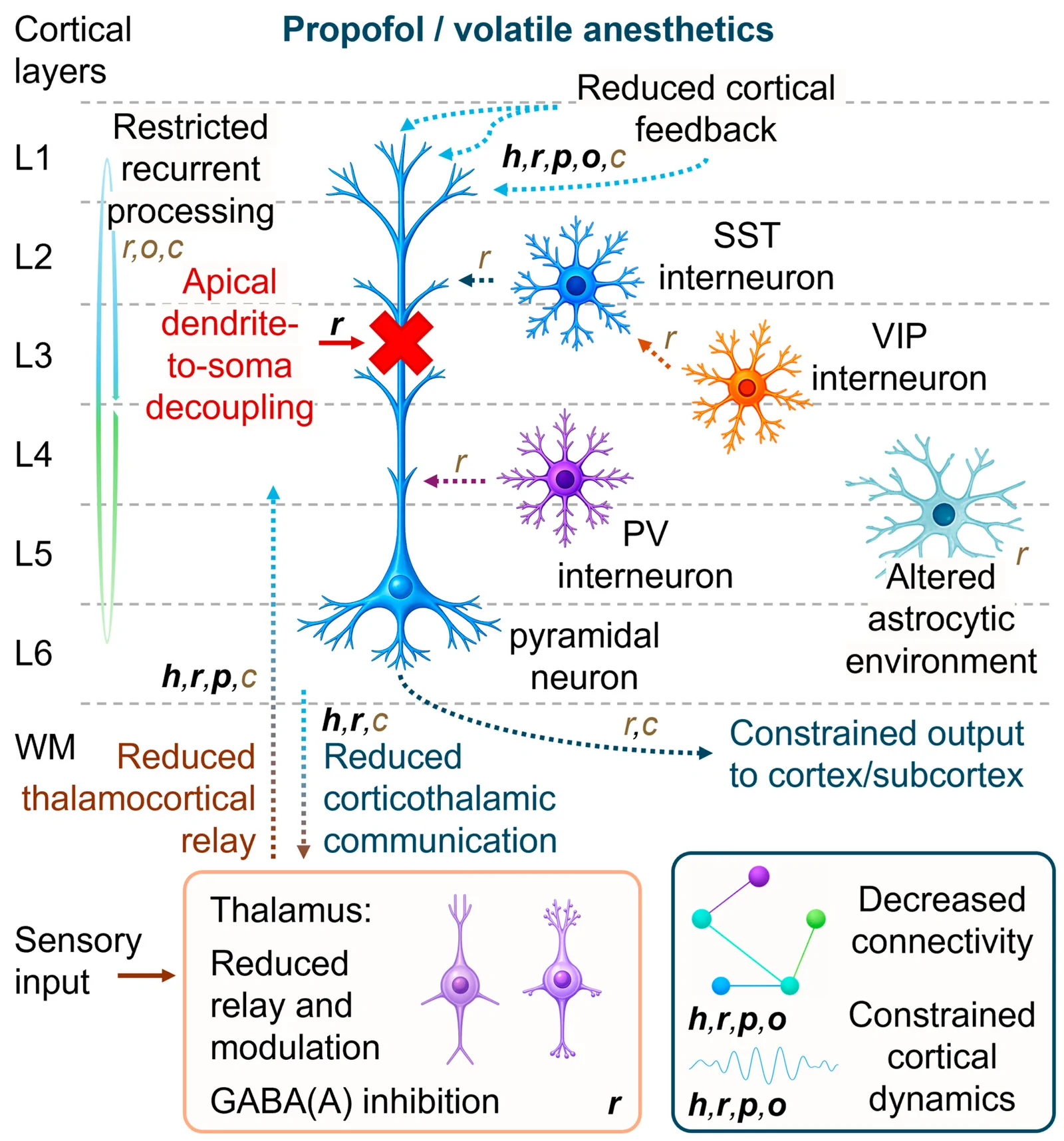
Restrictive cellular-to-network configuration under propofol and volatile anesthetics. Propofol and volatile anesthetics constrain cortical information processing at multiple interacting levels. Reduced influence of apical dendritic input on somatic output produces apical dendrite-to-soma decoupling, accompanied by reduced cortical feedback integration and restricted recurrent processing. Altered regulation by somatostatin-expressing (SST), vasoactive intestinal peptide-expressing (VIP), and parvalbumin-expressing (PV) interneurons may limit the flexibility of cortical microcircuits. Thalamic γ-aminobutyric acid type A receptor [GABA(A)] inhibition contributes to reduced thalamic relay and modulation, while thalamocortical relay and corticothalamic feedback are reduced. Changes in the astrocytic environment may additionally affect neuronal and network function. Together, these effects constrain cortical and subcortical output, decrease network connectivity, and restrict cortical dynamics. The relative contribution of individual mechanisms may differ among anesthetic agents, concentrations, brain regions, and experimental conditions. The figure is a conceptual representation and does not depict a complete wiring diagram. Evidence codes identify the source and level of support for individual elements: bold italic black codes indicate direct experimental support for the labeled process, whereas ochre italic codes indicate indirect or partial support, including inference from related measurements or models. Arrow colors correspond to the depicted pathways and cellular elements and do not indicate evidence strength. The reduced thickness of the multicolored recurrent loop represents restricted recurrent processing across cortical layers. *h*, human studies; *r*, rodent studies; *p*, non-human primate studies; *o*, studies in other species; *c*, computational or theoretical studies; L1–L6, cortical layers 1–6; WM, white matter; SST, somatostatin-expressing interneuron; VIP, vasoactive intestinal peptide-expressing interneuron; PV, parvalbumin-expressing interneuron; GABA(A), γ-aminobutyric acid type A receptor.

Classical anesthetics restrict several properties at once: the time available for neuronal firing, the spatial propagation of local activity, the diversity of responses to stimulation, and the set of cortical states through which the brain can move. Synchrony itself is not uniformly increased or decreased. Its distribution changes across frequencies, regions, and phases of the anesthetic state. The resulting activity remains structured, but it becomes less able to form differentiated and flexibly coordinated patterns across the cortex. Figure 2 summarizes this predominantly restrictive configuration, in which local activity may persist while recurrent processing, feedback integration, thalamic relay, cortical connectivity, and cortical/subcortical output become constrained.

## 4. Feedback, Sensory Hierarchies, and Thalamocortical State Control

The cellular effects described in the preceding sections acquire wider significance when they alter communication between cortical areas and between the cortex and thalamus. An early sensory response may remain detectable while its progression into association cortex is weakened or lost. Feedback pathways, higher order thalamic projections, and recurrent communication are especially relevant at this stage because they allow local responses to influence activity beyond the region in which they first arise.

### 4.1. Altered Feedback, Predictive Coding, and Sensory Hierarchy

Directional-connectivity studies show that feedback communication is particularly vulnerable during anesthesia. In 18 surgical patients, frontal-to-parietal feedback connectivity was stronger than parietal-to-frontal feedforward connectivity during wakefulness. After induction with propofol or sevoflurane, feedback connectivity weakened and the difference between the two directions became smaller. The wakeful pattern reappeared after responsiveness returned [47]. Ketamine produced a comparable directional change despite a different spectral profile. Alpha power decreased and gamma power increased, yet frontal-to-parietal feedback weakened after patients became unresponsive, while feedforward connectivity remained relatively preserved [48]. Similar behavioral endpoints can consequently be accompanied by different oscillatory changes and a shared reduction in feedback communication.

Experiments targeting individual pathways locate part of this vulnerability within cortical and thalamocortical afferents. In rat auditory cortex and mouse brain slices, responses carried by core thalamocortical pathways were less sensitive to isoflurane than responses conveyed through corticocortical and matrix thalamocortical pathways. At a concentration sufficient to abolish the righting reflex, auditory responses were preserved or enhanced, while visually driven top-down responses in auditory cortex were markedly reduced [49]. Direct optogenetic comparison of four afferent systems in mouse non-primary neocortex yielded a similar result. Corticocortical feedback responses were more sensitive to isoflurane than thalamocortical core, thalamocortical matrix, or corticocortical feedforward responses [50].

The frequency ranges carrying directional communication can also be affected unequally. Simultaneous recordings from peripheral and central structures in the fly brain showed that low-frequency activity between 0.1 and 5 Hz carried feedback from higher to lower levels, while activity between 10 and 45 Hz carried feedforward signaling in the opposite direction. Isoflurane preferentially reduced the low-frequency feedback component and left the higher-frequency feedforward component relatively intact [51]. The same general distinction has therefore been observed in mammalian cortical pathways and in a much smaller hierarchically organized nervous system.

Auditory paradigms reveal which responses persist as feedback signaling weakens. Intracranial recordings from human auditory cortex, auditory association regions, and prefrontal cortex examined responses to local and global deviations in sound sequences. Local deviance responses remained in most regions during responsive sedation but, after loss of responsiveness, were retained mainly within auditory cortex. Responses to global deviations were already strongly reduced during sedation and were absent after loss of responsiveness [52]. The initial detection of an acoustic irregularity can survive longer than the activity required to represent its relation to a wider sequence.

Recordings obtained during gradually increasing propofol exposure provide a related anatomical gradient. Propofol produced an increasing impairment along the auditory hierarchy: primary cortex retained field and spiking responses after unresponsiveness, but higher-order regions did not show comparable preservation. Cortical responses visible at the surface became restricted largely to auditory areas instead of extending into association cortex [53]. In macaques, auditory stimuli remained decodable in the auditory cortex under propofol, but stimulus synchronization between regions disappeared, and responses in the association and cognitive cortex became weak or absent. Even during cortical Up states, spiking responses were reduced in the auditory cortex and minimal at higher levels [54].

After propofol exposure, stimulus predictability no longer shaped sensory alpha- and beta-band activity, and communication in these bands between sensory and frontal regions weakened. Local responses to deviant sounds persisted, but their representation did not propagate reliably into higher-order cortex [55]. The hierarchy of spontaneous activity is altered as well. Resting-state fMRI in awake and anesthetized macaques showed that anesthesia reduced intrinsic ignition, a measure reflecting the capacity of local events to recruit distributed activity. Spatial and temporal aspects of cortical hierarchy changed differently and depended on anesthetic depth and the agent used. Lower ignition values were associated with a more rigid pattern of activity that followed structural connectivity more closely [56]. Hierarchical disruption is consequently visible even in the absence of an external stimulus. Propofol redistributed connectivity toward slower frequencies, increasing delta- and theta-band links while reducing connections from the alpha through gamma ranges. Preservation of parietal alpha connectivity was most closely associated with continued responsiveness [57]. The relevant change was not a uniform loss of connectivity, but a redistribution across frequencies and anatomical systems.

Across these studies, early sensory responses were often preserved after activity in association cortex, feedback pathways, or higher-order representations had declined. Sensory detection and conscious access should not be treated as the same event. The latter depends on whether local responses can continue through recurrent, predictive, and hierarchically organized pathways.

### 4.2. Thalamocortical Communication and Cortical Rhythms

Thalamocortical circuits connect cellular integration with distributed cortical state. Their role cannot be reduced to a single thalamic switch. Individual nuclei differ in their cortical projections, receptor composition, oscillatory behavior, and capacity to regulate arousal or sensory transmission.

Computational models link alpha activity during propofol anesthesia to changes within thalamocortical loops. Propofol can place these loops in a regime of persistent and synchronous alpha oscillations, producing cortical synchrony that may interfere with responses to external stimuli [58]. A model of alpha anteriorization separated the disappearance of posterior alpha from the appearance of frontal alpha. Reduced HCN current silenced thalamocortical cells proposed to support posterior alpha, while stronger GABAergic inhibition generated an alpha-timescale rhythm in reciprocal frontal thalamocortical circuits [59]. The anterior shift in alpha activity can thus arise from different effects in thalamic systems projecting to posterior and frontal cortex.

Human EEG-fMRI recordings show that internal thalamocortical activity can continue after the system has become less responsive to sensory input. In 16 participants, slow-wave activity increased after behavioral responses were lost and then reached an individual plateau despite increases in propofol concentration. Simultaneous EEG and fMRI in 12 participants showed that the thalamocortical system became isolated from external stimulation around this plateau, although internal exchange between the thalamus and cortex persisted [60]. Continued communication within the loop did not imply continued access to the environment. The timing of changes differs across anesthetics. During natural sleep onset and propofol anesthesia in rats, changes in high-frequency activity appeared first in the central medial thalamus and later in neocortex. Dexmedetomidine produced more simultaneous changes in the two regions, together with an abrupt shift in low-frequency phase relations at the loss of the righting reflex [61]. Thalamic and cortical changes therefore need not follow the same temporal order under different drugs.

Direct thalamic and cortical recordings during propofol administration identified coherent alpha activity between the thalamus and medial prefrontal cortex. At deeper levels, coherent delta oscillations developed alongside slower activity. The thalamic–prefrontal oscillatory pattern did not retrace its induction trajectory during recovery, indicating a different temporal organization of emergence [62]. Human recordings from the ventral intermediate thalamus and sensorimotor cortex provide an important qualification. Propofol increased local alpha power in both regions, but alpha and low-beta coherence between them declined. Coupling between the thalamic alpha phase and cortical gamma amplitude was also reduced [63]. Greater alpha power within two structures does not necessarily indicate stronger communication between them.

Propofol reorganized alpha activity between two anatomically distinct thalamocortical systems. Coupling weakened in a posterior, sensory network but became more coherent in prefrontal circuitry associated with cognitive thalamic nuclei, including the mediodorsal nucleus [64]. Functional-gradient mapping in healthy volunteers reached a related conclusion at a larger scale. Deep propofol sedation shifted thalamocortical organization toward a geometry deficient in connections to the transmodal cortex, and the magnitude of this shift was related to the distribution of matrix cells across the thalamus [65]. Thalamic involvement differed between the examined states. Changes during propofol anesthesia were concentrated around the pulvinar, while sleep recruited a different combination of first- and higher-order nuclei. Higher-order thalamic systems showed the most consistent involvement across conditions [66].

Arousal circuits can act on the thalamus through defined inhibitory pathways. GABAergic neurons in the lateral hypothalamus strongly inhibited thalamic reticular nucleus GABAergic neurons in mice: activation of this pathway produced rapid arousal during non-rapid-eye-movement sleep and sustained cortical arousal during deep anesthesia. Silencing the same connection increased the duration of non-rapid-eye-movement sleep and the amplitude of delta oscillations [67]. Suppressing GABAergic neurons in the thalamic reticular nucleus can therefore disinhibit thalamocortical activity during sleep and anesthesia. Receptor mechanisms within an individual nucleus can also influence the behavioral state. Lesioning the central medial thalamus shortened the loss of the righting reflex produced by gaboxadol or diazepam in rats. Local injection of gaboxadol, which preferentially activates extrasynaptic GABA(A) receptors, produced anesthesia and increased delta activity in medial prefrontal cortex; local diazepam, a positive allosteric modulator of benzodiazepine-sensitive GABA(A) receptors, did not produce the same effect. Reduced expression of the GABA(A)-receptor δ subunit within the central medial thalamus decreased sensitivity to both agents [68]. Extrasynaptic inhibition in this nucleus contributes to anesthesia, but its role varies depending on the drug’s pharmacological action.

Central thalamic stimulation demonstrates that selected nuclei can reorganize cortical activity. During propofol anesthesia in macaques, cortical and thalamic neurons became locked into slow Up and Down states, spiking declined, and coherence above 4 Hz decreased. Stimulation of the central thalamus restored wakeful behavior and reversed these electrophysiological changes [69]. In another nonhuman-primate experiment, central thalamic stimulation produced arousal and increased fMRI activity in the prefrontal, parietal, and cingulate cortices. It also restored a more varied repertoire of spontaneous activity, and reinstated hierarchical responses to auditory violations. Stimulation of the ventrolateral thalamus did not produce comparable effects [70].

Biophysical and cross-frequency models connect these systems-level findings with pyramidal-cell integration. In a network of layer 5 pyramidal neurons containing separate apical and somatic compartments, integrated information was greatest when diffuse thalamic input drove time-varying synchronous bursts. This regime combined widespread correlations with variable spiking and signatures of criticality [71]. Electrophysiological data from humans, mice, and rats identified a corresponding cross-frequency channel. Low-frequency activity between approximately 1 and 13 Hz in the cortex or thalamus was encoded by high-gamma activity in the other region. Propofol reduced this exchange, while 5-methoxy-N,N-dimethyltryptamine (5 mg/kg, subcutaneous) increased low-to-high frequency corticothalamic information transfer in five C57BL/6 mice [72]. Thalamocortical communication depends not only on whether two regions remain active, but also on whether activity in one can modulate the timing and frequency structure of the other.

### 4.3. Asymmetric Transitions Between Loss and Recovery of Responsiveness

Induction and emergence do not follow identical paths through neural state space. Drug concentration constrains both transitions, but it does not fully determine their timing or neural organization. Behavioral recovery involves changes in cortical processing speed, thalamocortical communication, ion regulation, arousal circuits, and sensory gating. Experiments in flies and mice established a conserved hysteresis between induction and emergence. Concentrations associated with entry into the anesthetized state differed from those associated with recovery. Mutations affecting sleep and wake regulation narrowed or widened this separation without corresponding changes in anesthetic uptake or metabolism [1]. Human EEG recordings reveal structure within both transitions. During gradual propofol induction, participants stopped responding to less salient clicks before they stopped responding to verbal stimuli. During emergence, responses to verbal stimuli returned before responses to clicks. Loss of responsiveness was accompanied by increased power below 1 Hz, disappearance of coherent occipital alpha, and development of coherent frontal alpha. A specific relation between low-frequency phase and alpha amplitude predicted recovery [2].

Functional imaging shows a clearer difference between the two directions. During gradual propofol induction, temporal autocorrelation increased across the brain, while corticocortical and subcorticocortical connectivity declined. Recovery was marked by an abrupt restoration of cortical processing timescales and a rapid increase in communication between subcortical structures and cortex. Pharmacokinetic modeling did not explain the difference between the two trajectories [73]. Entry into and exit from unresponsiveness were associated with distinct changes in processing speed and network interaction. The final transition into unresponsiveness can itself contain an ordered sequence. A hippocampal-thalamic-medial prefrontal pathway showed transiently increased synchrony followed by sequential deactivation around behavioral loss of responsiveness. Broader cortical activation developed only during the subsequently maintained unresponsive state [74]. Maintenance of an anesthesia-like state can also depend on a sharply localized brainstem population. Exposure of approximately 1900 or fewer neurons to muscimol within a small hotspot of the mesopontine tegmental anesthesia area was sufficient to sustain general anesthesia. Moving the injection 0.5 mm away removed the effect. The responsive cells were located within the reticular formation, in an area crossed by fibers of the superior cerebellar peduncle, and responded to propofol and pentobarbital [75]. A limited brainstem population can therefore exert effects that extend to cortical, motor, and sensory components of the anesthetized state.

Recovery recruits active molecular mechanisms within the thalamus. In mice exposed to several anesthetics, KCC2 was rapidly downregulated in the ventral posteromedial nucleus during emergence. Fbxl4 promoted the ubiquitination and degradation of KCC2, which reduced chloride extrusion, altered signaling through GABA(A) receptors, disinhibited thalamic neurons, and accelerated recovery of their excitability [76]. A subsequent study identified two parallel mechanisms initiated by EphB1 receptors in glutamatergic neurons of the same nucleus. EphB1 signaling increased NR2B phosphorylation and independently promoted KCC2 degradation. These changes activated the projection from glutamatergic neurons of the ventral posteromedial thalamus to glutamatergic neurons in the primary somatosensory cortex [77]. The paraventricular thalamus provides another route to recovery. A low dose of esketamine shortened the time to emergence from isoflurane anesthesia in mice and increased activity in glutamatergic neurons of the paraventricular thalamus. Chemogenetic suppression of this population abolished the effect of esketamine [78]. Astrocytes within the same nucleus can modify the process. Activation of paraventricular thalamic astrocytes, or reduction in astrocytic Kir4.1 expression, accelerated recovery from sevoflurane anesthesia, and the electrophysiological and single-cell analyses linked the effect mainly to a population of glutamatergic neurons projecting to the medial prefrontal cortex [79].

Behavioral recovery does not immediately normalize sensory filtering. During remimazolam sedation and anesthesia in mice, spontaneous activity and responses to paired tones were suppressed in the posterior parietal cortex, dorsal hippocampus, and mediodorsal thalamus. Recovery was followed by rebound activity and impaired auditory gating in all three regions. Top-down input returned towards baseline, while bottom-up auditory input exceeded baseline. The proportion and firing strength of thalamic reticular neurons responding to paired tones were reduced, weakening inhibition of repeated sensory input [80]. The early recovery state can therefore combine restored arousal with excessive sensory gain.

Electrical stimulation confirms that emergence depends on the state and location of the stimulated thalamic network. Stimulation of the intralaminar thalamus in macaques identified a network capable of producing rapid arousal during slow-wave activity. Genes enriched in the stimulation-responsive network were dominated by potassium-channel functions and mapped preferentially to excitatory cells, PV neurons, and oligodendrocytes [81]. Across propofol, sevoflurane, and ketamine anesthesia, stimulation of the central thalamus also reversed the increased reliance of distributed cortical activity on structural connectivity and restored several dimensions of cortical hierarchy, as well as behavioral arousal. Stimulation of the ventral lateral thalamus did not produce the same changes [82].

Loss and recovery of responsiveness are active transitions through partly different neural configurations. Induction can involve sequential deactivation, weakened feedback, altered thalamocortical rhythms, and recruitment of localized brainstem circuitry. Emergence requires the restoration of cortical and subcortical interactions, changes in thalamic excitability, the recruitment of specific projection systems, and the gradual normalization of sensory filtering. Recovery cannot be understood as the passive reversal of every process observed during induction.

## 5. Ketamine and Dissociative Reorganization

Ketamine provides a different route to anesthetic unresponsiveness. Propofol and volatile agents usually constrain cortical communication through slow oscillations, synaptic inhibition, and weakened feedback. Under ketamine, substantial activity can remain, but its distribution across cells, frequencies, and regions is reorganized. The state also changes with dose. Complex cortical responses and internally generated experiences may persist after behavioral responses are lost, while deeper anesthesia brings stronger slow activity and lower complexity.

### 5.1. Ketamine as a Contrasting Anesthetic Model

A direct comparison of propofol, xenon, and ketamine shows why behavioral unresponsiveness alone is insufficient to characterize the state induced by ketamine. Although all three drugs abolished responsiveness, their TMS-evoked patterns differed: propofol responses were local and simple, xenon responses were widespread but repetitive, and ketamine responses retained wake-like spatiotemporal diversity. Only the ketamine condition was followed by reports of prolonged internally generated experience [8]. Behavioral response, cortical complexity, and later reports of conscious content did not change together.

Ketamine can nevertheless share selected network endpoints with classical anesthetics. In surgical patients, frontal-to-parietal feedback connectivity declined after the loss of responsiveness, while feedforward connectivity remained relatively preserved. The spectral change differed from that observed with propofol or sevoflurane: alpha power decreased, while gamma power increased [48]. Functional MRI also showed that resting-state networks were affected unevenly. Connectivity within the default mode and salience networks was disrupted, while executive control, auditory, sensorimotor, and visual networks were affected much less [83]. Reduced feedback and altered higher-order connectivity can coexist with preserved activity in sensory systems.

Brain-wide c-Fos mapping showed that ketamine recruited cortical and subcortical systems spanning sensory, motor, affective, and reward functions. Isoflurane produced a different distribution, with greater representation of hypothalamic and homeostatic systems [84]. Intracortical recordings in macaques also revealed a gradual evolution of activity under ketamine. Loss and recovery of responsiveness occurred during progressive shifts between beta and gamma frequencies, with slow oscillations appearing later at deeper levels of anesthesia. Somatosensory responses remained detectable, while auditory and bimodal responses were more strongly reduced [85]. Ketamine does not impose the same change on every sensory pathway or cortical level.

Even the familiar EEG effects of ketamine can be separated into distinct components. When a subanesthetic infusion was given during general anesthesia, the increase in beta–gamma power remained, but the theta augmentation seen during awake ketamine administration was absent [86]. The oscillatory profile cannot be treated as a single signature. Some components persist during general anesthesia, while others depend on the surrounding brain state.

### 5.2. NMDA Antagonism, Interneurons, and Cortical Disinhibition

NMDA receptor antagonism is central to ketamine pharmacology. It does not, however, account for the full cellular response. Ketamine also acts on HCN channels and changes the participation of pyramidal neurons, interneurons, and recurrent cortical circuits. Such effects can preserve or amplify selected forms of activity while disrupting the organization needed for stable communication with the environment.

HCN1 provides a non-NMDA target for ketamine. At clinically relevant concentrations, suppression of I_h_ through HCN1 channels was accompanied by hyperpolarization and stronger coupling between dendrites and soma in cortical pyramidal cells, while global deletion of *Hcn1* reduced hypnotic sensitivity [87]. Selective deletion of *Hcn1* from excitatory forebrain neurons removed the corresponding membrane effects and shifted the ketamine concentration required for loss of the righting reflex upward by approximately 31% [88]. Forebrain principal neurons are one substrate through which HCN1 inhibition contributes to ketamine hypnosis.

At the population level, ketamine changes which neurons are active. In mouse neocortex, neurons with spontaneous activity before drug administration were often suppressed, while previously silent neurons became active. The switch occurred across cortical layers and regions and depended on NMDA receptors, HCN channels, and reduced activity of PV and SST interneurons [89]. A stable mean firing rate would miss this replacement of one active population by another. Two-photon imaging at matched sedative depth showed the same problem from a cell-type perspective. Isoflurane, propofol, and ketamine all reduced average activity in excitatory and inhibitory populations, but ketamine increased activity in a larger subset of excitatory neurons and preferentially activated a subset of SST interneurons [25].

Recurrent excitation adds a laminar mechanism. In the auditory cortex of gerbils, ketamine increased the response to pure tones within granular layers III and IV. The gain increase reflected stronger recurrent excitation around the thalamocortical input, along with a prolonged period of excitation caused by the stimulus [90]. In the retrosplenial cortex, ketamine reduced PV-interneuron activity while strengthening 1–3 Hz activity and delta-gamma coupling. Manipulation of the same cells reproduced or attenuated parts of the oscillatory and behavioral phenotype, with both NR1 and HCN1 contributing to the response [91].

Human electrophysiology and modeling support a wider contribution of inhibitory subtypes. Subanesthetic ketamine shifted human EEG spectra toward a flatter aperiodic component and stronger gamma activity, with the clearest effects over prefrontal and central regions. The spatial pattern corresponded to the expression of genes encoding parvalbumin (PV) and GluN2D. In a cortical model, reduced NMDA receptor activity in PV or SST interneurons increased gamma power by increasing pyramidal cell firing, although it did not reproduce the change in the aperiodic component [92]. Gamma enhancement captures one part of the cortical reorganization, not the entire shift in excitation and inhibition.

Ketamine also weakens the processing of unexpected sensory events. In awake mice, a deviant sound evoked a biphasic response in a subset of primary auditory neurons. Subanesthetic ketamine abolished the second peak, reduced responses to deviants in posterior parietal cortex, and impaired functional connectivity between auditory and parietal areas [93]. The impairment was visible at the level of individual neurons, a higher-order cortical region, and communication between the two areas. Under propofol, local deviance responses could persist within auditory cortex after loss of responsiveness, while global deviance responses and propagation to higher-order cortex were strongly reduced [52,55]. Comparable deviance paradigms were not examined for volatile anesthetics, precluding a direct comparison across agents.

### 5.3. Oscillatory Dynamics, Cortical Complexity, and Thalamocortical Activity

The complexity observed under ketamine changes with depth and cannot be interpreted without the accompanying behavioral and regional state. Perturbational complexity describes the diversity and spatial distribution of the brain response evoked by direct stimulation. In macaque ECoG, propofol sharply reduced neuronal avalanche size and duration, and decreased dynamical complexity. Neuronal avalanches are cascades of population activity that develop across successive time intervals. Ketamine allowed more wake-like avalanche dynamics to persist [12]. In rats, the perturbational complexity index changed little at the lowest dose sufficient to abolish behavioral response but fell at twice that dose. The deeper state was associated specifically with a lower high-to-low frequency ratio in posteromedial cortex [94]. Preserved complexity is most evident at lower levels of ketamine anesthesia and is not guaranteed to persist as the dose increases.

In the same experimental comparison, EEG measures of criticality distinguished unresponsiveness with and without reported experience and covaried with individual perturbational complexity [14]. Increasing ketamine exposure progressively increased dynamical stability, which describes how readily population activity returns toward its previous state after a perturbation, particularly at higher frequencies. This stabilization followed the decline in environmental interaction more closely than the inferred capacity for conscious experience [95]. Criticality, stability, and perturbational complexity describe related but non-equivalent features.

Communication between cortical regions can fail even when primary sensory activity remains. Primary sensory coding remained detectable in macaques, but ketamine reduced directed information transfer between anatomically connected cortical recording sites [96]. In the rat somatosensory system, ketamine increased spontaneous thalamic gamma power but reduced gamma activity evoked by sensory stimulation. The sensory gamma response was associated with the corticothalamic component of the thalamic potential, not with the earlier prethalamic component [97]. Ketamine can increase ongoing high-frequency activity while weakening the corticothalamic organization of sensory responses.

Posteromedial and retrosplenial circuits provide a more localized signature. Deep layer 5 cells in the retrosplenial cortex developed a 1–3 Hz rhythm after exposure to ketamine or phencyclidine. The activity was linked to selected thalamic targets but segregated from much of the brain; driving it optogenetically produced dissociation-like behavior, while its pharmacological generation required local HCN1 channels. A similar posteromedial signal accompanied self-reported dissociation in a patient with epilepsy [98]. Intracranial recordings in humans combined prefrontal and hippocampal gamma activity with an approximately 3 Hz signal in the posteromedial cortex. Adding propofol allowed the investigators to distinguish NMDA disinhibition from effects potentially shared through HCN1 [99].

**Figure 3:**
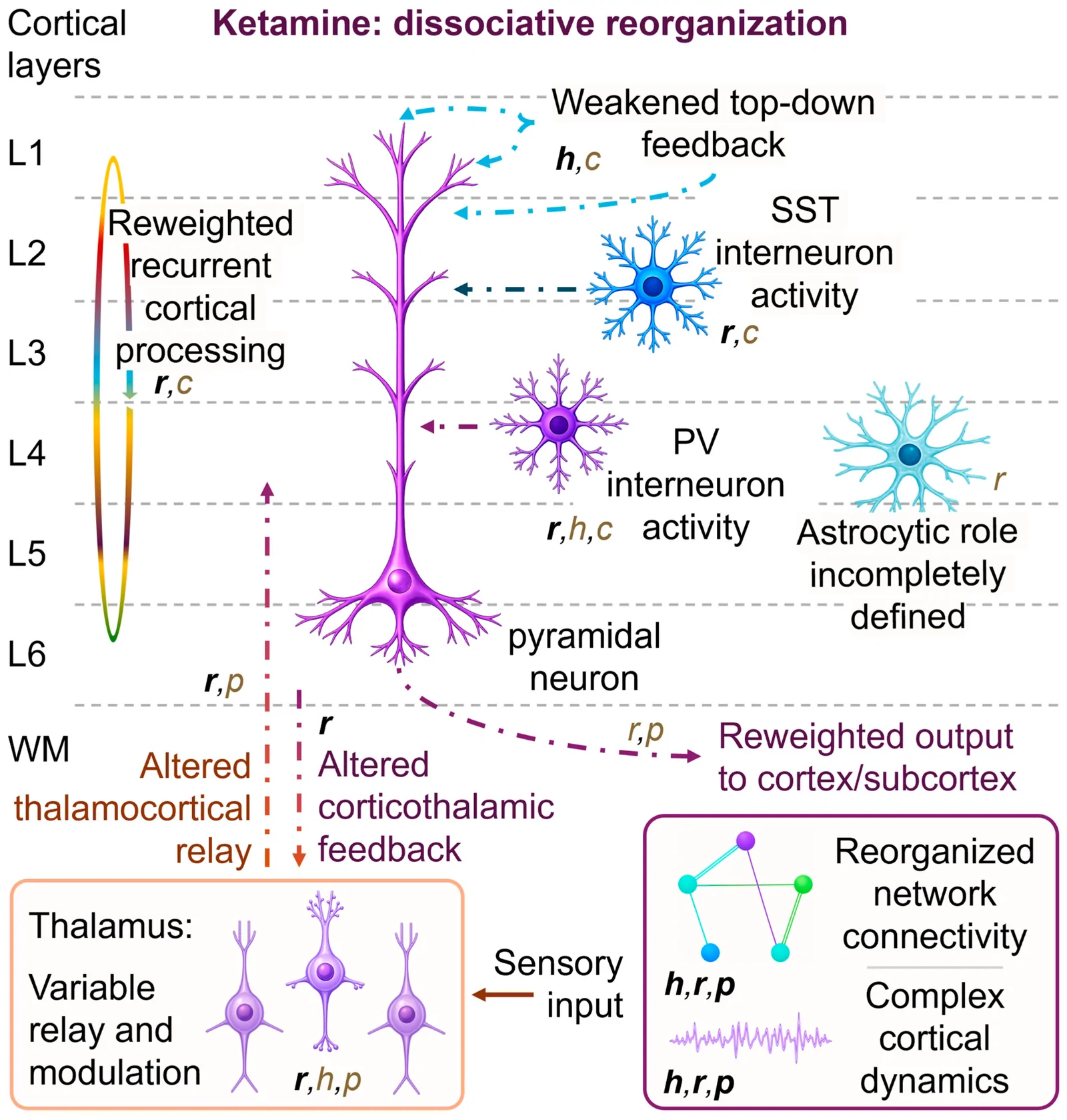
Dissociative cellular-to-network reorganization under ketamine. Ketamine reorganizes cortical and thalamocortical processing without producing the predominantly restrictive configuration associated with propofol and volatile anesthetics. Local recurrent cortical processing is reweighted, and top-down feedback to apical dendrites is weakened. The multicolored recurrent loop represents irregular and heterogeneous reweighting across cortical layers, rather than preservation of a continuous integration gradient. Changes in interactions among pyramidal neurons and somatostatin-expressing (SST), vasoactive intestinal peptide-expressing (VIP), and parvalbumin-expressing (PV) interneurons may alter the excitation-inhibition balance and local circuit gain. Thalamic relay and modulation remain variable, while thalamocortical relay, corticothalamic feedback, and cortical or subcortical output are altered or reweighted. Network connectivity is reorganized, and cortical dynamics may remain complex despite reduced responsiveness and impaired integration with the environment. The contribution of astrocytes remains incompletely defined. The figure is a conceptual representation and does not depict a complete wiring diagram. Evidence codes identify the source and level of support for individual elements: bold italic black codes indicate direct experimental support for the labeled process, whereas ochre italic codes indicate indirect or partial support, including inference from related measurements or models. Arrow colors distinguish pathway classes or the associated cell type and do not indicate evidence strength. *h*, human studies; *r*, rodent studies; *p*, non-human primate studies; *c*, computational or theoretical studies; L1–L6, cortical layers 1–6; WM, white matter; SST, somatostatin-expressing interneuron; VIP, vasoactive intestinal peptide-expressing interneuron; PV, parvalbumin-expressing interneuron.

A cortical model of ketamine action [100] showed that ketamine altered NMDA-receptor channel kinetics, disproportionately reducing tonic interneuron activity and producing disinhibition. The interaction between excitation mediated by NMDA-receptor and inhibition mediated by GABA-receptor generated gamma oscillations. At greater simulated doses, slow-delta events periodically interrupted the faster rhythm. Faster rhythms also arise through more than one circuit. In freely moving rats, ketamine increased neocortical gamma while suppressing gamma and generating coherent 130–180 Hz oscillations in the olfactory bulb. Local blockade showed that the high-frequency oscillation depended on kainate receptors and intact GABA(A) transmission and propagated through olfactory–limbic pathways, but did not account for neocortical gamma [101]. Figure 3 summarizes this dissociative reorganization, in which recurrent processing, regulation by interneurons, top-down feedback, thalamocortical coupling, network connectivity, and cortical dynamics are reweighted or altered, while substantial activity and relatively high complexity may persist.

### 5.4. Dissociation Between Responsiveness, Connectedness, and Conscious Content

Ketamine shows why an absent behavioral response cannot by itself establish an absent experience. In the TMS-EEG comparison, participants were unresponsive under ketamine but later reported vivid experiences, and the evoked cortical response remained complex [8]. Resting-state EEG from the same experimental contrast placed ketamine closer to critical dynamics associated with retained consciousness than propofol or xenon, despite the common behavioral endpoint [14].

A retrospective connectome harmonic analysis of resting-state fMRI data from eight healthy volunteers compared wakefulness with a state of unresponsiveness induced by ketamine. Fine-grained spatial harmonics contributed more strongly during ketamine unresponsiveness. The resulting pattern aligned with connectome-harmonic signatures of psychedelic states induced by LSD or subanesthetic ketamine and diverged from patterns observed during propofol anesthesia or in unresponsive patients with disorders of consciousness after brain injury [102]. At the group level, the analysis identified a neural organization compatible with disconnected consciousness despite the absence of a behavioral response.

Thalamic metabolism also depends on the anesthetic used. In a randomized study of propofol, dexmedetomidine, sevoflurane, and S-ketamine, lower thalamic glucose metabolism distinguished connected from disconnected states for the first three agents, but not for S-ketamine. Drug exposure produced wider cortical changes than the state contrast itself [11]. A marker that separates connectedness under one anesthetic class may not generalize to ketamine.

Ketamine can uncouple behavioral responsiveness from environmental connectedness and from the presence of reportable, internally generated experience. Not every unresponsive state under ketamine should be treated as conscious: deeper doses reduce complexity and introduce slow activity. Interpretation depends on dose, regional dynamics, sensory coupling, and the method used to infer experience. Whether an experience is later available for report also depends on the memory processes considered in the next section. The principal cellular and circuit differences between classical anesthetics and ketamine, together with their functional implications, are summarized in Table 1.

## 6. Memory, Reportability, and the Limits of Behavioral Inference

The preceding sections considered anesthetic states in terms of cellular integration, cortical communication, thalamocortical regulation, and dissociative reorganization. Memory introduces a separate interpretive problem. A postoperative report depends not only on the state present during anesthesia, but also on whether information was retained until it could be tested. Conversely, evidence of implicit memory indicates that information influenced later performance, but does not establish that it formed part of conscious experience. Behavioral response, environmental connectedness, memory, and later report should consequently be examined as related but separate observations. Studies of amnesia involving specific receptors, isolated forearm responses, serial awakenings, hippocampal activity, and postanesthetic sensory processing show that these observations can diverge through identifiable neural mechanisms.

### 6.1. Explicit Recall, Connectedness, and Behavioral Inference

Etomidate enhanced tonic inhibition through GABA(A) receptors that contain the α5 subunit and weakened long-term potentiation in CA1. Spatial and nonspatial memory impairment was absent in α5-null mice, whereas sedation, motor impairment, and loss of the righting reflex remained comparable to wild-type animals. The α5-containing receptors, therefore, contributed selectively to the amnestic rather than the principal hypnotic effects of etomidate [7].

Motor unresponsiveness has its own limitations. A meta-analysis of the isolated forearm technique included 22 studies and 1131 patients. An isolated forearm response occurred in 393 patients during induction or maintenance of general anesthesia. Responses were reported less frequently during induction than during maintenance, but estimates varied across anesthetic regimens, and the included studies showed substantial heterogeneity and generally low methodological quality [106]. The result does not establish preserved connected consciousness in every clinically unresponsive patient. It shows that the absence of a conventional motor response can fail to detect command processing when neuromuscular blockade prevents movement in the rest of the body. Across 61 studies involving 3906 patients and 119 cohorts, implicit-memory effects were observed in 43 of those cohorts. They were more likely in patients with ASA physical status III–IV and under general anesthesia than deep sedation, but less likely after benzodiazepine premedication. Such effects demonstrate a later influence of information presented during anesthesia, but cannot establish that it was consciously experienced at the time [107].

Serial-awakening studies separate environmental connectedness from the presence of experience more directly. During dexmedetomidine sedation, propofol sedation, and natural sleep, participants were awakened repeatedly and asked about their experiences during the preceding period. EEG source localization analysis identified different signatures for sensory disconnection and unconsciousness. Disconnection was associated with spatially extensive spectral changes, while unconsciousness was linked more specifically to reduced activity in the anterior and posterior cingulate cortices. A signature derived from dexmedetomidine data showed partial generalization to propofol sedation and sleep [9].

None of the ten candidate resting-state EEG measures uniquely separated the presence from the absence of experience. Several were related instead, or additionally, to environmental connectedness; frontal-to-posterior normalized symbolic transfer entropy tracked connectedness across propofol, dexmedetomidine, and sleep. Permutation entropy and the spectral exponent declined when disconnected experience gave way to no reported experience [10]. An EEG change observed in an unresponsive person may consequently reflect environmental disconnection, loss of experience, or both.

The distinction also depends on the anesthetic. In a randomized study of 160 healthy men, connected and disconnected states were compared during propofol, dexmedetomidine, sevoflurane, and S-ketamine exposure. Reduced thalamic glucose metabolism was associated with disconnectedness under propofol, dexmedetomidine, and sevoflurane, but not under S-ketamine. Drug exposure itself produced widespread cortical metabolic suppression that was more extensive than the difference between connected and disconnected participants [11].

Explicit recall, implicit memory, motor response, environmental connectedness, and conscious experience do not provide interchangeable descriptions of an anesthetic state. Each depends on a different set of experimental observations. Later recall remains clinically important, but its absence cannot identify whether the earlier failure concerned conscious processing, memory formation, or access to the external environment.

### 6.2. Hippocampal Mechanisms, Consolidation, and Postanesthetic Continuity

The studies in this section address different stages at which later memory can be altered. In adult mice, isoflurane, medetomidine combined with midazolam and fentanyl, and ketamine combined with xylazine all reduced CA1 spiking, decorrelated cellular ensembles, and altered dendritic spine dynamics. CA1 activity under each regimen differed from natural sleep. Network changes resolved more readily after isoflurane than after the other two protocols. Memory consolidation was impaired after ketamine combined with xylazine and after medetomidine combined with midazolam and fentanyl, but not after isoflurane [103]. Reduced CA1 activity alone did not predict the memory outcome. Subhypnotic propofol altered communication between the hippocampus and precuneus during a semantic auditory task in 25 volunteers. Reduced coupling involved several hippocampal subfields and was associated with subsequent memory performance despite preserved behavioral responsiveness [104]. Hippocampal communication may thus weaken at an amnestic dose that leaves behavioral responsiveness intact.

In neuronal and astrocytic cocultures, enhancement of tonic currents through GABA(A) receptors remained detectable 24 h after exposure to etomidate or sevoflurane. Ketamine blocked this persistent response through a mechanism involving BDNF–TrkB and GSK-3β signaling, limited the surface accumulation of α5-containing receptors, and reduced later recognition and spatial-memory deficits after sevoflurane in mice [105]. Molecular effects continuing after anesthetic exposure may therefore influence memory after behavioral recovery. Return of arousal does not immediately restore normal sensory processing. Recovery from remimazolam was accompanied by rebound activity and weaker paired-tone gating across the posterior parietal cortex, dorsal hippocampus, and mediodorsal thalamus. Prefrontal input approached baseline while ascending auditory input became excessive. The number and activity of the tone-responsive GABAergic neurons of the thalamic reticular nucleus decreased [80].

The evidence discussed above points to anesthesia influencing later memory by altering hippocampal activity during recovery. A report obtained after awakening reflects the combined effects of these processes and does not directly establish whether experience was present during anesthesia.

## 7. Neuron–Glia Interactions and Emerging Mechanisms

### 7.1. Astrocytes

Astrocytic calcium signaling was more sensitive to anesthesia than the corresponding neuronal sensory response. Ketamine/xylazine, isoflurane, and urethane suppressed spontaneous and stimulus-evoked calcium events, as well as those associated with arousal, in neocortical astrocytes, even when whisker-evoked neuronal activity remained measurable [17]. Removing astrocytic *Ndufs4* had little effect on the anesthetic concentration required for induction but altered emergence: righting and tail-clamp responses returned at lower isoflurane or halothane concentrations. This recovery phenotype occurred without a measurable change in total brain norepinephrine [108]. Related work links mitochondrial complex I to anesthetic sensitivity beyond astrocytes. Complex I defects altered volatile-anesthetic sensitivity in *C. elegans* and mice, while isoflurane inhibition of complex I reduced presynaptic ATP availability and disrupted excitatory vesicle recycling in mouse hippocampal slices [109,110,111]. These findings connect mitochondrial function with synaptic transmission and anesthetic endpoints, but they do not establish one mitochondrial mechanism shared by all anesthetics. Activation of parabrachial astrocytes promoted wakefulness, reduced isoflurane sensitivity, and accelerated emergence. During anesthesia, it also reduced cortical delta power and burst suppression, whereas inhibition of these cells shifted these measures in the opposite direction [112]. Reversible remodeling of fine astrocytic processes during inhaled anesthesia depended on the phosphorylation state of Ezrin. Disrupting this mechanism reduced astrocyte–synapse contact, increased sevoflurane sensitivity, and was accompanied by stronger tonic GABAergic inhibition and lower pyramidal-cell excitability [113]. Ezrin links the astrocytic membrane to the actin cytoskeleton. Separate studies indicate that anesthetic compounds can alter microtubule stability, while stabilization of microtubules with epothilone B delays loss of the righting reflex during isoflurane anesthesia in rats and mice [114,115,116]. A biophysical analysis relating tubulin oscillations to anesthetic potency remains theoretical [117]. This literature identifies microtubules as potential anesthetic targets but does not show that they mediate the astrocytic remodeling involving Ezrin described here.

Sleep and sevoflurane shared selected changes in astrocytic proteins. Their overall proteomic and phosphorylation profiles remained distinct. The effects of anesthesia involved structural regulation, metabolism, and molecular clearance, together with reduced NUCKS1-Ser188 phosphorylation and changes in signaling, mRNA processing, and developmental pathways [118]. The studies concern different cellular processes and different brain regions. They do not point to one common astrocytic mechanism.

### 7.2. Glial Effects in Thalamic and Arousal Circuits

Within the paraventricular thalamus, activating astrocytes or reducing astrocytic Kir4.1 accelerated recovery from sevoflurane anesthesia, and single-cell and electrophysiological analyses localized this effect mainly to PVT^ChAT^ neurons projecting to the medial prefrontal cortex, and not to the PVT^GRM^ population [79]. Removing microglia shifted both anesthetic transitions toward a shorter anesthetized state, delaying induction and hastening emergence—involving P2Y12 calcium signaling and regionally divergent changes in neuronal activity instead of altered dendritic-spine plasticity [119]. These findings show that astrocytes and microglia can modify anesthetic sensitivity, induction, and emergence through distinct effects on neuronal activity. Representative glial mechanisms affecting anesthetic sensitivity, state stability, and emergence are summarized in Table 2.

## 8. Cellular-to-System Model

The findings reviewed above do not form a single sequence leading from drug binding to behavior. Molecular targets establish the initial conditions, but the resulting state depends on how their effects are distributed across dendritic compartments, local cortical circuits, thalamocortical pathways, glial cells, and memory systems. Similar behavioral endpoints can emerge from different configurations of these processes. An account of anesthesia must consequently describe not only what activity is lost, but also how the remaining activity is organized.

### 8.1. Two Modes of Anesthetic Disruption

Propofol and volatile anesthetics most often constrain the routes through which local activity influences wider cortical processing. At the thalamic level, sensory relay can be weakened through shunting inhibition or loss of sustained responses. Within the cortex, distal apical input becomes less effective in driving pyramidal-cell output, layer 5 activity becomes abnormally synchronized, and neuronal firing is restricted to limited phases of slow activity [5,15,16,29,30]. Each mechanism reduces the range over which a local response can affect subsequent processing.

The same restriction appears across the sensory hierarchy. Feedback pathways are more sensitive to anesthesia than many feedforward pathways, and frontal-to-parietal feedback declines after loss of responsiveness [47,49,50]. Early auditory responses and information about stimulus identity may remain detectable, while activity in association and cognitive regions becomes weak or absent. Predictive modulation and communication between sensory and frontal cortex are also lost before all local sensory responses disappear [53,54,55]. Sensory input can reach the cortex without acquiring the distributed influence associated with conscious access. Propofol does not abolish thalamocortical activity—frontal alpha rhythms may become more prominent even as communication with posterior and transmodal regions weakens [63,64,65]. Strong rhythmic activity can coexist with reduced communication across the wider system.

In the studies reviewed here, classical anesthetics predominantly restrict access to wider processing. Neurons may still fire, low-level sensory pathways may remain responsive, and parts of the large-scale network structure may persist, but the remaining activity is nevertheless constrained by altered dendritic coupling, recurrent processing, feedback, and thalamocortical timing. The capacity of local events to recruit differentiated and coordinated responses across the wider system is reduced.

Ketamine reorganizes cortical activity instead of suppressing it uniformly, by changing which neuronal populations remain active and altering the contribution of interneuron subtypes [25,89,91]. Recurrent excitation may also increase in selected cortical circuits [90]. HCN1 inhibition provides another cellular mechanism [87]. Posteromedial and retrosplenial circuits illustrate this reorganization. Ketamine can produce a low-frequency rhythm in deep retrosplenial neurons that is coupled to selected thalamic regions and separated from much of the remaining brain. Related posteromedial activity has been observed during human dissociation [98,99]. Gamma activity may also increase through several cortical and subcortical mechanisms. Strong ongoing activity may coexist with impaired transmission of sensory information between cortical regions [92,96,97,101].

Behavioral unresponsiveness under ketamine can coexist with complex cortical responses and later reports of vivid internally generated experiences [8]. Neuronal avalanche dynamics remain more wake-like than under propofol, although this preservation is not independent of dose. Deeper ketamine anesthesia reduces perturbational complexity and introduces stronger slow activity [12,94]. Increasing dissociative effects have also been associated with greater stability of cortical activity, particularly at higher frequencies, even when measures related to criticality remain compatible with conscious experience [95].

The distinction between suppression and dissociative reorganization is a working hypothesis, not an established taxonomy. Ketamine can weaken frontal-to-parietal feedback [48], and classical anesthetics can produce several internally structured states instead of one uniform suppression. The two modes describe the dominant organization of activity. Under propofol and volatile agents, local responses lose access to wider processing. Under ketamine, activity may remain substantial but becomes organized in a way that weakens normal contact with the environment. Xenon is a boundary case. Although xenon and ketamine both inhibit NMDA receptors, xenon produced widespread but repetitive TMS-evoked activity and no later report of experience at the tested unresponsive endpoint, whereas ketamine retained differentiated responses and vivid reports [8]. NMDA-receptor antagonism alone does not determine restrictive or dissociative organization. Dexmedetomidine provides a second boundary case. It reduced thalamic metabolism during disconnectedness, as did propofol and sevoflurane, but showed a different timing of thalamic and cortical changes [11,61]. It is better described as a predominantly restrictive or disconnected state reached through a distinct arousal mechanism, not as a fixed member of one molecular class.

### 8.2. Separable Outputs of Anesthetic State Organization

Responsiveness, environmental connectedness, conscious content, memory, and later report are related observations, but they do not measure the same process. A motor response requires perception of the command, sufficient arousal, selection of an action, and an available motor pathway. Connectedness requires ongoing sensory events to influence the current experience. Later report additionally depends on memory mechanisms operating during and after the event.

Clinical unresponsiveness has limited resolution when motor output is constrained. Isolated forearm responses have been observed in a subset of patients during general anesthesia, despite the absence of a conventional motor response from the paralyzed body [106]. Such responses do not establish that connected consciousness is common in all unresponsive patients. They show that behavioral silence cannot identify the stage at which command processing failed. Environmental connectedness and conscious experience can also separate. Serial-awakening studies during sedation and sleep identified partly different EEG patterns for sensory disconnection and the absence of reported experience. Measures previously interpreted as markers of consciousness were often associated with connectedness as well, and none of the examined resting-state measures was specific only to conscious experience [9,10]. Reduced thalamic metabolism distinguished connected from disconnected states under propofol, dexmedetomidine, and sevoflurane, but not under S-ketamine [11]. A physiological marker of connectedness under one drug class may not generalize to another.

Ketamine makes this distinction particularly visible. Unresponsive participants may later describe vivid experiences unrelated to the environment, and connectome harmonic analysis places unresponsiveness during ketamine administration closer to some altered conscious states than to propofol anesthesia or unconsciousness after brain injury [8,102]. Conscious experience may remain present even when its relation to ongoing sensory events and overt behavior is weakened. Memory provides an additional axis. Amnesia induced by etomidate can be reduced by deleting GABA(A) receptors that contain the α5 subunit, without producing an equivalent reduction in its sedative and hypnotic effects [7,103]. Different anesthetic regimens also produce different effects on hippocampal ensemble organization, dendritic spine dynamics, and memory consolidation despite comparable behavioral suppression [103]. Propofol can alter connectivity between the hippocampus and precuneus at an amnestic but subhypnotic concentration [104], while receptor changes affecting tonic inhibition may persist after the anesthetic has been removed [105].

Evidence of implicit memory shows only that earlier information influenced later performance. It does not reveal whether the information was consciously experienced when presented [107]. Explicit recall has a different limitation. Its absence can reflect anesthetic effects on hippocampal activity, functional connectivity, memory consolidation, or persistent receptor regulation [7,103,104,105]. Neither endpoint provides a direct record of the experience itself.

A postoperative report is a compound outcome. It reflects the conscious content that may have been present, the degree of connection to the environment, and whether the event remained available in memory after recovery. Failure at any of these levels can result in an event being removed from the final report. The clinical terms conscious and unconscious conceal these distinctions when inferred solely from behavior or recall.

### 8.3. Cellular Mechanisms and High-Dimensional State Models

No single measurement captures all dimensions of an anesthetic state. Firing rate describes the output of a neuronal population but not how its activity is distributed across cells or cortical regions. Spectral power identifies prominent rhythms but cannot determine whether those rhythms support effective communication. Functional connectivity does not specify which cellular mechanisms produced the observed relation. Complexity measures add information about differentiation and temporal structure, but their interpretation also depends on the anesthetic, dose, behavioral task, and definition of consciousness.

Studies of ongoing and perturbed activity show why several dimensions are needed. Measures of criticality derived from spontaneous EEG can predict individual perturbational complexity, but unresponsive states differ according to whether conscious experience is retained [14]. Lempel-Ziv and statistical complexity can change in opposite directions among people exposed to the same propofol protocol, with the initial state influencing later responsiveness [42]. Deep anesthesia itself contains several electrocortical configurations, including states with unexpectedly high complexity, while burst and suppression periods recruit different distributed neuronal ensembles [43,44].

Dynamic stability adds another dimension. Propofol, ketamine, and dexmedetomidine all prolong the return of cortical population activity after sensory perturbation despite their different molecular actions [46]. A shared behavioral endpoint may contain both cellular effects that differ among drugs and common changes in population dynamics. Neither level should be used as a substitute for the other. Glial cells also modify the anesthetic state. Changes in astrocytic or microglial function can affect anesthetic sensitivity and the course of emergence [17,79,108,113,119].

A cellular-to-system model should describe the joint configuration of dendritic coupling, pyramidal and interneuron activity, recurrent cortical processing, thalamic and corticocortical communication, glial regulation, hippocampal function, and population dynamics, and should distinguish among responsiveness, connectedness, memory, reportability, and conscious content. The purpose of such a model is not to identify one final mechanism of anesthesia or consciousness. It is to explain how different cellular perturbations generate distinct brain states, how those states change during induction and recovery, and why similar outward behavior can accompany different forms of neural organization. The operational distinctions required to interpret these multidimensional anesthetic states are summarized in Table 3.

### 8.4. Limitations and Unresolved Questions

This review used one database, included only English-language records, and applied a publication-date limit beginning in 2005. One author conducted title and abstract screening, and no independent duplicate assessment or formal risk-of-bias ranking was performed. The synthesis combines cellular preparations, animal models, human recordings, randomized studies, and meta-analyses that differ in species, anesthetic, dose, exposure duration, depth, and operational endpoint. Behavioral unresponsiveness, motor suppression, electrophysiological changes, and later report do not provide equivalent measures of conscious experience, and cross-species mechanistic extrapolation remains limited.

Several questions remain unresolved. It is not known how presynaptic, cytoskeletal, and mitochondrial targets generate cell-type selectivity, which mechanisms govern induction and emergence, or when astrocytic changes cause a state transition instead of modifying anesthetic sensitivity and recovery. Methods are also needed that distinguish responsiveness, connectedness, and experience at the level of an individual subject.

## 9. Conclusions

General anesthesia rarely silences the brain. Local sensory responses, neuronal firing, oscillations, and parts of large-scale organization can remain after responsiveness has disappeared. What changes most clearly is the remaining activity’s ability to coordinate across cortical layers and distant regions.

With propofol and volatile agents, communication becomes increasingly restricted. Apical input exerts less influence on pyramidal-cell output, recurrent cortical processing narrows, feedback weakens, and thalamocortical exchange becomes less flexible. Ketamine can produce a similar outward loss of responsiveness through a different neural state. Substantial activity may persist, but its organization no longer supports normal contact with the environment. Internally generated content may continue, and neither behavior nor later recall reliably reflects its presence.

These observations limit what can be inferred from clinical signs alone. An absent motor response does not reveal whether the failure occurred during command processing, conscious access, or motor execution. Missing postoperative recall is equally ambiguous. Anesthesia can alter hippocampal activity, functional connectivity, memory consolidation, and receptor regulation after exposure. None of these findings, considered alone, demonstrates that experience was absent.

## Figures and Tables

**Table 1:** Cellular and circuit mechanisms linking anesthetic action to conscious-state organization.

Organizational Level and Principal Substrate/Process	Propofol, Volatile Anesthetics, and Other Anesthetic Regimens	Ketamine	Functional Relevance
Synaptic and receptor-level transmission Phasic and tonic currents through GABA(A) receptors, glutamatergic transmission, membrane conductance, and the contribution of HCN1 channels to I_h_.	Propofol strengthens inhibitory shunting through GABA(A) receptors in thalamic relay cells. Volatile agents and etomidate reduce sustained responses in relay and pyramidal cells through distinct effects on excitatory conductances and on synaptic and extrasynaptic inhibition [29,30,31,32].	NMDA-receptor antagonism is accompanied by HCN1 inhibition. The resulting hyperpolarization and altered dendrite-to-soma coupling do not correspond to a uniform increase in inhibition [87,88].	Changes the probability and timing with which local inputs generate neuronal output and enter thalamocortical or cortical processing.
Apical dendrite-to-soma integration Integration of basal sensory input with distal apical feedback, contextual input, higher-order thalamic input, and neuromodulation in pyramidal neurons.	General anesthesia can uncouple distal apical activity from somatic firing. Effective coupling in the awake state also depends on neuromodulatory and higher-order thalamic support [15].	HCN1 inhibition can strengthen dendrite-to-soma coupling in cortical pyramidal cells, even though communication across the wider cortical system is reorganized [87].	Local sensory activity may persist without the contextual amplification needed to influence distributed conscious processing.
Layer 5 pyramidal output and ensemble recruitment Intrinsic excitability, repetitive firing, long-range pyramidal output, and the identity of neurons participating in the active ensemble.	Layer 5 pyramidal activity becomes abnormally synchronized across cortical distances, while repetitive firing and pyramidal-cell excitability can decline. Increasing local activity does not necessarily restore behavioral responsiveness [16,38,39,40].	Previously active neurons may be suppressed while previously silent neurons are recruited. Selected excitatory and SST-interneuron populations can increase their activity despite reduced population averages [25,89].	Mean firing rate can conceal changes in ensemble membership, temporal flexibility, and the capacity of pyramidal output to carry differentiated information.
Interneurons and excitation-inhibition organization PV, SST, and VIP interneuron activity; glutamatergic drive onto excitatory and inhibitory cells; and control of cortical gain and rhythms specific to cell-type.	Isoflurane does not simply increase inhibition: cortical GABA transmission can decline, PV activity can become suppressed and synchronized, and VIP changes can appear later. Different agents produce distinct interneuron configurations at similar behavioral depth [24,25].	Reduced PV-interneuron activity contributes to retrosplenial low-frequency activity and behavior related to dissociation. NMDA-receptor hypofunction in PV or SST interneurons can increase gamma activity through greater pyramidal-cell firing [25,91,92].	Anesthetic states reflect reorganization of excitation and inhibition across cell types, rather than movement along a single global E/I axis.
Recurrent cortical processing Plateau potentials, sustained spiking, recurrent excitation, and local amplification after thalamocortical input.	Propofol suppresses muscarinic plateau potentials and sustained spiking, limiting the cellular basis of persistent recurrent activity [26].	The effects of ketamine on sustained activity depend on dose, and ketamine can enhance recurrent excitation during thalamocortical input processing in granular layers of the auditory cortex [26,89,90].	Classical agents can truncate sustained processing, while ketamine can preserve or amplify selected local activity without ensuring stable communication with the environment.
Corticocortical feedback and sensory hierarchy Feedback and feedforward pathways, predictive modulation, sensory-to-association propagation, and communication between the sensory and frontal cortex.	Feedback pathways are generally more sensitive than feedforward pathways. Early sensory responses can remain after prediction modulation, association-cortical responses, and sensory-frontal communication have weakened [47,49,50,53,55].	Frontal-to-parietal feedback also declines after unresponsiveness. Local responses can persist despite impaired deviance processing and reduced communication between the sensory and higher-order cortex [48,93].	Detection of a stimulus can be separated from its recurrent, predictive, and distributed processing associated with conscious access.
Thalamic relay and thalamocortical state control Sensory relay, higher-order nuclei, thalamocortical phase relations, cortical coupling of individual nuclei, and central-thalamic control of arousal.	Propofol weakens relay and reorganizes alpha activity between posterior sensory and prefrontal networks. Higher-order nuclei are repeatedly implicated, and central-thalamic stimulation can restore cortical dynamics and behavior in experimental models [29,62,64,66,69,70].	Ongoing thalamic gamma can increase while stimulus-evoked corticothalamic gamma declines. A retrosplenial low-frequency rhythm can couple to selected thalamic targets while becoming segregated from much of the rest of the brain [97,98].	Persistent activity within thalamocortical loops does not, by itself, demonstrate sensory access; the nucleus, direction, timing, and cortical target remain critical.
Oscillatory timing and spike-field coordination Slow oscillations, alpha anteriorization, gamma activity, cross-frequency coupling, and the phase windows available for neuronal firing.	Slow activity restricts spiking to narrow temporal windows and can fragment coordination across regions. Frontal alpha can increase as posterior alpha and effective thalamocortical communication decline [2,5,41,62].	Beta-gamma activity can remain prominent, while deeper exposure introduces slow activity. Posteromedial low-frequency rhythms and gamma activity can coexist in regionally segregated configurations [85,99,100].	Spectral power alone cannot establish effective communication; the spatial distribution, phase relationship, and coupling of rhythms determine their functional significance.
Complexity, criticality, and dynamical stability Perturbational complexity, neuronal avalanches, critical dynamics, network efficiency, state repertoire, and recovery after perturbation.	Propofol and volatile anesthesia generally reduce differentiated responses and constrain network transitions, although several low-complexity and higher-complexity configurations can occur at the same drug concentration [3,13,14,42,46].	Avalanche and perturbational dynamics resembling wakefulness can persist near the first loss of responsiveness. As exposure increases, complexity declines, slow activity becomes stronger, and dynamical stability increases [12,14,46,94,95].	Unresponsiveness is not equivalent to low complexity. Interpretation requires the anesthetic dose, baseline state, region, and distinction between spontaneous and evoked measures.
Hippocampal and memory mechanisms Hippocampal ensemble activity, alpha5-containing GABA(A) receptors, functional connectivity, consolidation, persistent tonic inhibition, and sensory gating during recovery.	Etomidate separates amnesia from sedation through α5-containing GABA(A) receptors. Remimazolam alters auditory gating during recovery. Isoflurane and combined regimens produce distinct hippocampal and memory effects. Subhypnotic propofol alters hippocampal connectivity, while GABA(A) receptor changes can persist after etomidate or sevoflurane [7,80,103,104,105].	Ketamine can prevent persistent changes in tonic GABA(A) inhibition and later memory deficits after sevoflurane [105].	Later recall reflects encoding, consolidation, retrieval, and recovery-state processing; it is not a direct record of whether experience was present during anesthesia.

Note: References are representative and not exhaustive. Functional relevance represents an interpretive synthesis of the evidence cited in the agent columns; references are not repeated in the final column. Effects vary with agent, dose, brain region, species, and experimental endpoint. GABA(A), gamma-aminobutyric acid type A receptor; HCN1, hyperpolarization-activated cyclic nucleotide-gated channel 1; I_h_, hyperpolarization-activated current; PV, parvalbumin; SST, somatostatin; VIP, vasoactive intestinal peptide.

**Table 2:** Glial mechanisms modifying anesthetic sensitivity, state stability, and emergence.

Glial Substrate or Process	Experimental Evidence	Effect on the Anesthetic State	Interpretive Relevance
Cortical astrocytic Ca^2+^ signaling	In mice with restrained heads, ketamine–xylazine, isoflurane, and urethane suppressed spontaneous and stimulus-evoked astrocytic Ca^2+^ activity [17].	Astrocytic signals were suppressed at doses that still permitted measurable neuronal sensory responses.	Neuronal responsiveness can persist despite marked astrocytic suppression; causality for unresponsiveness remains unproven.
Astrocytic mitochondrial complex I	Inducible deletion of astrocytic *Ndufs4* altered recovery from isoflurane and halothane more clearly than induction [108].	Behavioral responses returned at lower anesthetic concentrations in knockout mice.	Astrocytic metabolism may influence emergence independently of mechanisms governing induction.
Parabrachial astrocytes	Chemogenetic activation increased wakefulness, reduced isoflurane sensitivity, and shortened emergence; inhibition produced opposite electrophysiological effects [112].	Astrocytic activity modified cortical delta power, burst suppression, anesthetic sensitivity, and recovery.	Astrocytes in a nucleus involved in arousal can causally modify anesthetic depth and emergence.
Perisynaptic astrocytic processes and Ezrin	Inhaled anesthetics reversibly impaired fine astrocytic processes and altered Ezrin phosphorylation; experimental disruption of Ezrin function increased sevoflurane sensitivity [113].	Reduced astrocyte–synapse interactions accompanied stronger tonic GABAergic inhibition and lower pyramidal-cell excitability.	Structural astrocyte–synapse coupling may influence neuronal sensitivity to volatile anesthesia.
Paraventricular thalamic astrocytes and Kir4.1	Astrocyte activation or selective reduction in Kir4.1 expression promoted recovery from sevoflurane anesthesia [79].	The effect was mediated mainly through PVT^ChAT^ neurons, not the PVT^GRM^ population; PVT^ChAT^ neurons projected primarily to the medial prefrontal cortex.	Astrocytic potassium conductance may regulate emergence through a defined thalamocortical pathway.
Microglial P2Y12 signaling	Microglial depletion delayed induction and accelerated emergence; the effects depended on P2Y12 signaling and microglial Ca^2+^ influx [119].	Microglia influenced neuronal activity differently in regions associated with anesthesia and emergence.	Microglia may stabilize the anesthetic state, but the evidence does not identify one brain-wide mechanism.
Astrocytic proteomic regulation	Sleep and sevoflurane produced partly overlapping but distinct proteomic and phosphoproteomic changes in cortical astrocytes [118].	Anesthesia affected pathways related to structure, metabolism, molecular clearance, and synaptic signaling.	Molecular overlap with sleep is incomplete and does not imply a single shared astrocytic state.

Note: References are representative rather than exhaustive. Interpretive relevance summarizes the cited evidence; effects vary with anesthetic, dose, model, brain region, and experimental endpoint.

**Table 3:** Interpretive separation of responsiveness, connectedness, conscious experience, memory, and reportability.

Dimension and Operational Measure	Positive Evidence and What It Does Not Establish	Major Limitations
Behavioral responsiveness [2,8,10,106] Response to a verbal command or salient stimulus; isolated forearm response; righting reflex or other predefined motor endpoint in experimental animals.	PE: For command following, successful perception and sufficient processing of the instruction together with an available motor pathway. Simpler reflexes establish only preserved motor reactivity to the tested stimulus. NE: Absence of conscious experience, absence of sensory processing, or environmental disconnection.	Neuromuscular blockade, impaired motor execution, stimulus salience, fluctuating arousal, response criterion, and effects of individual drugs on motor systems.
Environmental connectedness [9,10,11,52] Immediate or serial-awakening report containing external events; command processing; neural evidence that ongoing sensory information reaches higher-order processing.	PE: External sensory events influenced the current experience or remained available for immediate report. NE: Absence of internally generated experience or complete absence of consciousness.	Sensory attenuation, disrupted feedback, reduced salience, impaired report, rapid forgetting, and differences among anesthetics in neural signatures of disconnection.
Presence of conscious experience [8,9,14,102] Immediate report after serial awakening; later report interpreted with memory limitations; convergent perturbational, EEG, or evidence from connectome analysis.	PE: At least some experiential content was present and remained accessible to the measurement method. NE: Absence of experience when report, encoding, retention, or retrieval may have failed.	Dependence on report, temporal delay, amnesia, reconstruction after awakening, uncertain thresholds for neural markers, and group-level rather than individual inference.
Conscious content [8,9,102] Description of internally generated or externally connected experience; report of specific content; neural patterns associated with dissociation or altered conscious states.	PE: The reported type or organization of experience, including whether it was internally generated or linked to the environment. NE: Absence of experience when no content is reported, or equivalence between reported content and the full experience that occurred.	Memory selection, verbal expressibility, reconstruction, demand characteristics, and weak correspondence between regional neural markers and specific phenomenology.
Explicit recall [7,103,104,105] Free recall, prompted recall, recognition, or a postoperative account of material presented during anesthesia.	PE: Information was encoded, retained through consolidation, retrieved after recovery, and available for overt report. NE: Absence of experience, environmental disconnection, or failure of sensory processing at the time of presentation.	Amnesia mediated by receptor, hippocampal ensemble disruption, impaired consolidation, altered hippocampal connectivity, retrieval failure, and postanesthetic receptor changes.
Implicit memory [107] Priming, altered task performance, or another later behavioral effect of information presented during anesthesia without explicit recollection.	PE: Earlier information affected later processing or behavior despite the absence of explicit recall. NE: Conscious perception at the time of presentation or subsequent availability of the information for report.	Heterogeneous paradigms, baseline response tendencies, anesthetic depth, clinical status, premedication, and difficulty separating perceptual from response-level priming.
Postanesthetic report and reportability [7,8,9,103,105] Retrospective verbal report obtained after recovery, including reports of external events, dreams, or internally generated experiences.	PE: Experiential content remained accessible after recovery and could be translated into a communicable response. NE: That no experience occurred, that the person was disconnected from the environment, or that sensory processing was absent.	All limitations affecting experience, connectedness, encoding, consolidation, retrieval, language, willingness to report, timing of questioning, and reconstruction after emergence.

Note: References are representative rather than exhaustive. PE, interpretation of positive evidence; NE, conclusions that cannot be drawn from a negative finding. Positive evidence is interpreted only for the operational measure used; no single behavioral, electrophysiological, imaging, memory, or endpoint based on report fully specifies an anesthetic state.

## Data Availability

No new data were created or analyzed in this study. Data sharing is not applicable to this article.

## Abbreviations

The following abbreviations are used in this manuscript:

BDNF	brain derived neurotrophic factor
c-Fos	cellular Fos proto-oncogene protein
ChAT	choline acetyltransferase
E/I	excitation/inhibition
ECoG	electrocorticography
EEG	electroencephalography
EphB1	Eph receptor B1
Fbxl4	F-box and leucine rich repeat protein 4
fMRI	functional magnetic resonance imaging
GABA	γ-aminobutyric acid
GABA(A)	γ-aminobutyric acid type A receptor
GluN2D	glutamate ionotropic receptor NMDA type subunit 2D
GRM	glutamate metabotropic receptor
GSK-3β	glycogen synthase kinase 3 beta
HCN	channel activated by hyperpolarization and gated by cyclic nucleotides
HCN1	HCN channel 1
I_h_	current activated by hyperpolarization
KCC2	potassium–chloride cotransporter 2
Kir4.1	inward rectifier potassium channel 4.1
Kv1.2	voltage gated potassium channel 1.2
L1–L6	cortical layers 1–6
NMDA	N-methyl-D-aspartate
NR1	NMDA receptor subunit 1, also termed GluN1
NR2B	NMDA receptor subunit 2B, also termed GluN2B
*Ndufs4*	NADH:ubiquinone oxidoreductase subunit S4
NUCKS1	nuclear casein kinase and cyclin dependent kinase substrate 1
P2Y12	P2Y purinergic receptor 12
PV	parvalbumin expressing interneuron
PVT	paraventricular thalamus
SST	somatostatin expressing interneuron
TMS	transcranial magnetic stimulation
TrkB	tropomyosin receptor kinase B
VIP	vasoactive intestinal peptide expressing interneuron
WM	white matter

## Appendix A

**Table A1:** Thematic PubMed search modules, exact queries, and record counts across the literature-selection process.

Search Module, Topic, and Number of PubMed Records	PubMed Query
S1. General anesthesia as a perturbation of consciousness; *n* = 524	((((general[tiab] AND (anesth*[tiab] OR anaesth*[tiab])) OR propofol[tiab] OR sevoflurane[tiab] OR isoflurane[tiab] OR ketamine[tiab] OR xenon[tiab] OR midazolam[tiab]) AND (consciousness[tiab] OR unconsciousness[tiab] OR unresponsiveness[tiab] OR connectedness[tiab] OR disconnectedness[tiab] OR “loss of consciousness”[tiab] OR “recovery of consciousness”[tiab]) AND (neurobiology[tiab] OR neurobiological[tiab] OR (neural[tiab] AND correlate*[tiab]) OR “neural mechanism”[tiab] OR “neural mechanisms”[tiab] OR “brain state”[tiab] OR “brain states”[tiab] OR “network state”[tiab] OR “network states”[tiab] OR “common endpoint”[tiab] OR “common end point”[tiab])) OR ((general[tiab] AND (anesth*[tiab] OR anaesth*[tiab])) AND (sleep[tiab] OR coma[tiab]) AND (consciousness[tiab] OR unconsciousness[tiab] OR arousal[tiab] OR “brain state”[tiab] OR “brain states”[tiab])) OR ((anesth*[tiab] OR anaesth*[tiab]) AND consciousness[tiab] AND (connectedness[tiab] OR disconnectedness[tiab])) OR ((anesth*[tiab] OR anaesth*[tiab]) AND consciousness[tiab] AND unconsciousness[tiab] AND (integration[tiab] OR disconnection[tiab] OR “information capacity”[tiab]))) AND 2005/01/01:2026/07/01[dp] AND english[la]
S2. Cellular, dendritic, pyramidal-neuron and synaptic mechanisms; *n* = 780	(((consciousness[tiab] OR “conscious processing”[tiab] OR “conscious perception”[tiab] OR “conscious state”[tiab]) AND (cellular[tiab] OR dendrit*[tiab] OR “apical dendrite”[tiab] OR “apical dendrites”[tiab] OR “apical amplification”[tiab] OR “pyramidal neuron”[tiab] OR “pyramidal neurons”[tiab] OR “layer 5”[tiab] OR L5[tiab]) AND (integration[tiab] OR coupling[tiab] OR decoupling[tiab] OR recurrent[tiab] OR arousal[tiab] OR “top-down”[tiab] OR “bottom-up”[tiab])) OR ((anesth*[tiab] OR anaesth*[tiab] OR propofol[tiab] OR isoflurane[tiab] OR sevoflurane[tiab] OR ketamine[tiab]) AND (dendrit*[tiab] OR “apical dendrite”[tiab] OR “apical dendrites”[tiab] OR “pyramidal neuron”[tiab] OR “pyramidal neurons”[tiab] OR “layer 5”[tiab] OR L5[tiab] OR interneuron*[tiab] OR GABAA[tiab] OR “GABA-A”[tiab] OR (“gamma-aminobutyric acid”[tiab] AND receptor*[tiab]) OR NMDA[tiab] OR “N-methyl-D-aspartate”[tiab] OR HCN1[tiab] OR synaptic[tiab] OR “excitatory-inhibitory”[tiab] OR “E/I”[tiab]) AND (consciousness[tiab] OR unconsciousness[tiab] OR “loss of consciousness”[tiab] OR “anesthetic-induced unconsciousness”[tiab] OR “anaesthetic-induced unconsciousness”[tiab] OR “hypnotic action”[tiab] OR “hypnotic actions”[tiab] OR “hypnotic effect”[tiab] OR “hypnotic effects”[tiab])) OR ((anesth*[tiab] OR anaesth*[tiab] OR propofol[tiab] OR etomidate[tiab] OR isoflurane[tiab] OR sevoflurane[tiab] OR halothane[tiab] OR xenon[tiab] OR ketamine[tiab]) AND (((SNARE[tiab] OR syntaxin*[tiab] OR synaptobrevin*[tiab] OR SNAP-25[tiab] OR “release machinery”[tiab] OR “neurotransmitter release”[tiab]) AND (bind*[tiab] OR inhibit*[tiab] OR sensitiv*[tiab] OR resist*[tiab] OR recovery[tiab])) OR ((microtubul*[tiab] OR tubulin[tiab] OR cytoskelet*[tiab] OR epothilone[tiab] OR taxane*[tiab]) AND (bind*[tiab] OR modulat*[tiab] OR stabil*[tiab] OR destabil*[tiab] OR sensitiv*[tiab] OR resist*[tiab] OR potency[tiab] OR unconsciousness[tiab] OR recovery[tiab] OR emergence[tiab] OR target*[tiab])) OR ((mitochondri*[tiab] AND (“complex I”[tiab] OR NDUFS4[tiab] OR “NADH dehydrogenase”[tiab])) AND (inhibit*[tiab] OR modulat*[tiab] OR sensitiv*[tiab] OR hypersensitiv*[tiab] OR synaptic[tiab] OR neurotransmitter*[tiab]))))) AND 2005/01/01:2026/07/01[dp] AND english[la]
S3. Propofol and volatile anesthetics: EEG, synchrony, feedback and predictive processing; *n* = 421	((propofol[tiab] OR sevoflurane[tiab] OR isoflurane[tiab] OR “volatile anesthetic”[tiab] OR “volatile anesthetics”[tiab] OR “volatile anaesthetic”[tiab] OR “volatile anaesthetics”[tiab]) AND (“loss of consciousness”[tiab] OR “recovery of consciousness”[tiab] OR “return of consciousness”[tiab] OR “loss of responsiveness”[tiab] OR unconsciousness[tiab] OR unresponsiveness[tiab] OR “altered state of consciousness”[tiab] OR “altered states of consciousness”[tiab] OR “conscious state”[tiab] OR “conscious states”[tiab] OR “consciousness state”[tiab] OR “consciousness states”[tiab] OR “states of consciousness”[tiab] OR connectedness[tiab] OR disconnectedness[tiab] OR disconnection[tiab] OR “state transition”[tiab] OR “state transitions”[tiab] OR “brain state”[tiab] OR “brain states”[tiab]) AND (connectivity[tiab] OR “functional connectivity”[tiab] OR “effective connectivity”[tiab] OR “directed connectivity”[tiab] OR “directed functional connectivity”[tiab] OR “brain network”[tiab] OR “brain networks”[tiab] OR “cortical network”[tiab] OR “cortical networks”[tiab] OR “large-scale brain network”[tiab] OR “large-scale brain networks”[tiab] OR corticocortical[tiab] OR “cortico-cortical”[tiab] OR thalamocortical[tiab] OR “thalamo-cortical”[tiab] OR corticothalamic[tiab] OR “cortico-thalamic”[tiab] OR “cortico-subcortical”[tiab] OR “core-matrix”[tiab] OR “basal forebrain”[tiab] OR “substantia innominata”[tiab] OR “parabrachial nucleus”[tiab] OR “ventral tegmental area”[tiab] OR “nucleus accumbens”[tiab] OR feedback[tiab] OR feedforward[tiab] OR “top-down”[tiab] OR frontoparietal[tiab] OR “frontal-parietal”[tiab] OR “cortical hierarchy”[tiab] OR “sensory processing”[tiab] OR “sensory signal”[tiab] OR “sensory signals”[tiab] OR “predictive coding”[tiab] OR “predictive routing”[tiab] OR “information flow”[tiab] OR “information transfer”[tiab] OR “information exchange”[tiab] OR “information integration”[tiab] OR “information processing”[tiab] OR complexity[tiab] OR criticality[tiab] OR “critical dynamics”[tiab] OR “integrated information”[tiab] OR (integration[tiab] AND segregation[tiab]) OR “brain dynamics”[tiab] OR “network dynamics”[tiab] OR microstate*[tiab] OR “EEG dynamics”[tiab] OR “electroencephalographic dynamics”[tiab] OR “EEG signature”[tiab] OR “EEG signatures”[tiab] OR “electroencephalogram signature”[tiab] OR “electroencephalogram signatures”[tiab] OR “electroencephalographic signature”[tiab] OR “electroencephalographic signatures”[tiab] OR “phase-amplitude”[tiab] OR “cross-frequency”[tiab] OR “spectral slope”[tiab] OR “spectral exponent”[tiab] OR aperiodic[tiab] OR “traveling wave”[tiab] OR “traveling waves”[tiab] OR “travelling wave”[tiab] OR “travelling waves”[tiab] OR decoupling[tiab])) AND 2005/01/01:2026/07/01[dp] AND english[la]
S4. Thalamocortical and corticothalamocortical mechanisms of loss and recovery; *n* = 245	((anesth*[tiab] OR anaesth*[tiab] OR propofol[tiab] OR sevoflurane[tiab] OR isoflurane[tiab] OR sedation[tiab]) AND (thalamocortical[tiab] OR thalamus[tiab] OR thalamic[tiab] OR corticothalamocortical[tiab] OR corticothalamic[tiab] OR “core-matrix”[tiab] OR “paraventricular thalamus”[tiab] OR brainstem[tiab]) AND (consciousness[tiab] OR unconsciousness[tiab] OR “loss of consciousness”[tiab] OR recovery[tiab] OR emergence[tiab] OR disconnectedness[tiab] OR transition*[tiab]) AND (connectivity[tiab] OR communication[tiab] OR network*[tiab] OR rhythm*[tiab] OR alpha[tiab] OR dynamics[tiab] OR “phase-amplitude”[tiab])) AND 2005/01/01:2026/07/01[dp] AND english[la]
S5. Ketamine, dissociation, cortical complexity and memory; *n* = 526	((ketamine[tiab] OR “S-ketamine”[tiab] OR esketamine[tiab] OR “R-ketamine”[tiab] OR arketamine[tiab]) AND (((dissociation[tiab] OR dissociative[tiab] OR “altered consciousness”[tiab] OR “alteration of consciousness”[tiab] OR consciousness[tiab] OR unconsciousness[tiab] OR unresponsiveness[tiab] OR “loss of consciousness”[tiab] OR “return of consciousness”[tiab] OR connectedness[tiab] OR disconnectedness[tiab] OR “sensory disconnection”[tiab]) AND (EEG[tiab] OR electroencephalograph*[tiab] OR MEG[tiab] OR magnetoencephalograph*[tiab] OR fMRI[tiab] OR “functional magnetic resonance”[tiab] OR phMRI[tiab] OR intracranial[tiab] OR ECoG[tiab] OR “local field potential”[tiab] OR LFP[tiab] OR “resting-state”[tiab] OR “resting state”[tiab] OR “functional connectivity”[tiab] OR “effective connectivity”[tiab] OR complexity[tiab] OR entropy[tiab] OR “signal diversity”[tiab] OR “spectral exponent”[tiab] OR “critical dynamics”[tiab] OR “brain dynamics”[tiab] OR “cortical dynamics”[tiab] OR microstate*[tiab] OR “connectome harmonic”[tiab] OR “information integration”[tiab] OR oscillat*[tiab] OR rhythm*[tiab] OR gamma[tiab] OR “high-frequency”[tiab] OR “phase-amplitude”[tiab] OR coactivation[tiab] OR “predictive coding”[tiab] OR thalamocortical[tiab] OR corticothalamic[tiab] OR corticocortical[tiab] OR posteromedial[tiab] OR retrosplenial[tiab] OR “default mode”[tiab] OR “salience network”[tiab])) OR ((anesthesia[tiab] OR anaesthesia[tiab] OR sedation[tiab] OR emergence[tiab] OR vigilance[tiab]) AND (unconsciousness[tiab] OR unresponsiveness[tiab] OR “loss of consciousness”[tiab] OR “return of consciousness”[tiab] OR consciousness[tiab] OR disconnectedness[tiab]) AND (EEG[tiab] OR electroencephalograph*[tiab] OR fMRI[tiab] OR “functional connectivity”[tiab] OR “effective connectivity”[tiab] OR complexity[tiab] OR “signal diversity”[tiab] OR “spectral exponent”[tiab] OR “brain dynamics”[tiab] OR “cortical dynamics”[tiab] OR oscillat*[tiab] OR gamma[tiab] OR “phase-amplitude”[tiab] OR thalamocortical[tiab] OR corticothalamic[tiab] OR corticocortical[tiab] OR posteromedial[tiab])) OR ((NMDA[tiab] OR “N-methyl-D-aspartate”[tiab] OR interneuron*[tiab] OR disinhibition[tiab] OR parvalbumin[tiab] OR GABAergic[tiab] OR GABAA[tiab] OR “GABA-A”[tiab] OR HCN1[tiab] OR acetylcholine[tiab]) AND (cortical[tiab] OR cortex[tiab] OR neocortex[tiab] OR thalamocortical[tiab] OR corticothalamic[tiab] OR corticocortical[tiab] OR posteromedial[tiab] OR retrosplenial[tiab] OR prefrontal[tiab]) AND (dissociation[tiab] OR dissociative[tiab] OR consciousness[tiab] OR unconsciousness[tiab] OR unresponsiveness[tiab] OR oscillat*[tiab] OR rhythm*[tiab] OR gamma[tiab] OR EEG[tiab] OR “functional connectivity”[tiab] OR “effective connectivity”[tiab] OR complexity[tiab] OR “brain dynamics”[tiab] OR “cortical dynamics”[tiab])) OR ((amnesia[tiab] OR recall[tiab] OR “explicit recall”[tiab] OR “implicit memory”[tiab] OR “episodic memory”[tiab] OR encoding[tiab] OR retrieval[tiab] OR “working memory”[tiab] OR “visual working memory”[tiab] OR P300[tiab] OR P3b[tiab] OR “mismatch negativity”[tiab]) AND (EEG[tiab] OR electroencephalograph*[tiab] OR ERP[tiab] OR “event-related potential”[tiab] OR fMRI[tiab] OR MEG[tiab] OR magnetoencephalograph*[tiab] OR intracranial[tiab] OR ECoG[tiab]) AND (dissociation[tiab] OR dissociative[tiab] OR consciousness[tiab] OR volunteer*[tiab] OR “healthy human”[tiab] OR “healthy humans”[tiab] OR primate*[tiab] OR macaque*[tiab] OR monkey*[tiab])))) AND 2005/01/01:2026/07/01[dp] AND english[la]
S6. Reportability, memory and connectedness under anesthesia; *n* = 442	((((explicit[tiab] AND recall[tiab]) OR (awareness[tiab] AND recall[tiab]) OR (implicit[tiab] AND memory[tiab]) OR (unconscious[tiab] AND memory[tiab]) OR ((connected[tiab] OR disconnected[tiab] OR connectedness[tiab] OR disconnectedness[tiab]) AND consciousness[tiab]) OR (isolated[tiab] AND forearm[tiab] AND technique[tiab]) OR ((amnesia[tiab] OR amnesic[tiab]) AND (explicit[tiab] OR implicit[tiab] OR recall[tiab] OR awareness[tiab])) OR ((dream*[tiab] OR dreaming[tiab]) AND recall[tiab])) AND (anesthesia[tiab] OR anaesthesia[tiab] OR anesthetic*[tiab] OR anaesthetic*[tiab] OR propofol[tiab] OR isoflurane[tiab] OR sevoflurane[tiab] OR etomidate[tiab] OR midazolam[tiab] OR remimazolam[tiab])) OR ((((connected[tiab] OR disconnected[tiab] OR connectedness[tiab] OR disconnectedness[tiab]) AND consciousness[tiab]) OR (isolated[tiab] AND forearm[tiab] AND technique[tiab]) OR (recall[tiab] AND awareness[tiab])) AND (intubation[tiab] OR tracheal[tiab] OR paralysis[tiab] OR paralysed[tiab] OR paralyzed[tiab]))) AND 2005/01/01:2026/07/01[dp] AND english[la]
S7. Astrocytic and glial modulatory mechanisms; *n* = 27	((anesthesia[tiab] OR anaesthesia[tiab] OR anesthetic*[tiab] OR anaesthetic*[tiab] OR propofol[tiab] OR isoflurane[tiab] OR sevoflurane[tiab] OR etomidate[tiab]) AND (astrocyt*[tiab] OR ((glia[tiab] OR glial[tiab]) AND (calcium[tiab] OR GABAA[tiab] OR “GABA-A”[tiab] OR mitochondri*[tiab] OR morpholog*[tiab] OR proteome[tiab] OR phosphorylation[tiab] OR glycogen[tiab] OR lactate[tiab] OR glymphatic[tiab])) OR ((astrocyt*[tiab] OR glia[tiab] OR glial[tiab]) AND (basal[tiab] AND forebrain[tiab])) OR ((astrocyt*[tiab] OR glia[tiab] OR glial[tiab]) AND parabrachial[tiab]) OR ((astrocyt*[tiab] OR glia[tiab] OR glial[tiab]) AND (paraventricular[tiab] AND thalamus[tiab])) OR ((astrocyt*[tiab] OR glia[tiab] OR glial[tiab]) AND (cingulate[tiab] OR prefrontal[tiab]))) AND (consciousness[tiab] OR unconsciousness[tiab] OR wakefulness[tiab] OR arousal[tiab] OR emergence[tiab] OR ((recovery[tiab] OR return[tiab]) AND consciousness[tiab]) OR (loss[tiab] AND consciousness[tiab]) OR (state[tiab] AND transition*[tiab]))) AND 2005/01/01:2026/07/01[dp] AND english[la]
Final combined search; *n* = 2401	S1 OR S2 OR S3 OR S4 OR S5 OR S6 OR S7
Title screening; *n* = 778	Titles of all 2401 records retrieved by the combined search were screened for indications that they addressed cellular, microcircuit, corticothalamic, thalamocortical, glial, memory, dissociative, or related mechanisms involved in anesthetic disruption of conscious-state organization. Records of uncertain relevance were retained to preserve sensitivity. Following title screening, 778 of the 2401 records were retained for abstract screening.
Abstract screening. Selected articles: *n* = 119	Abstracts were evaluated for direct mechanistic relevance to the review. Eligible studies included original experimental, human neurophysiological, animal, cellular, imaging, computational, and meta-analytic investigations providing mechanistic insight into anesthetic effects on consciousness, responsiveness, connectedness, memory, reportability, or cellular-to-system integration. Selected articles were included in the narrative synthesis.

Note: Searches were restricted to title and abstract fields and to English-language records published from 1 January 2005 to 1 July 2026, inclusive. Modules S1–S7 were combined using the OR operator for the final search. Module-level counts are not additive because individual records could be retrieved by more than one module; each record was counted once in the combined set. Counts in the final two rows indicate records retained after title and abstract screening. S1–S7, search modules 1–7; [tiab], title/abstract; [dp], publication date; [la], language; *n*, number of records; *, PubMed wildcard. The basal forebrain and ventral tegmental area terms included in S3 did not yield studies retained in the final narrative synthesis. The final search was run on 8 August 2026, during revision, when module S2 was modified to capture presynaptic release machinery, cytoskeletal and microtubule mechanisms, and mitochondrial complex I mechanisms.
